# The rice *EP3* and *OsFBK1* E3 ligases alter plant architecture and flower development, and affect transcript accumulation of microRNA pathway genes and their targets

**DOI:** 10.1111/pbi.13710

**Published:** 2021-10-01

**Authors:** Rita S. Borna, Erik H. Murchie, Kevin A. Pyke, Jeremy A. Roberts, Zinnia H. Gonzalez‐Carranza

**Affiliations:** ^1^ Plant and Crop Sciences Division School of Biosciences University of Nottingham Nottingham UK; ^2^ Present address: Department of Botany University of Dhaka Dhaka 1000 Bangladesh; ^3^ Present address: Faculty of Science and Engineering School of Biological & Marine Sciences University of Plymouth Devon UK

**Keywords:** rice, F‐box proteins, flower development, microRNA pathway

## Abstract

ERECTA PANICLE 3 (EP3) and ORYZA SATIVA F‐BOX KELCH 1 (OsFBK1) proteins share 57% and 54% sequence identity with the Arabidopsis F‐box protein HAWAIIAN SKIRT (HWS). Previously we showed that *EP3* is a functional orthologue of *HWS*. Here we demonstrate that *OsFBK1* is another functional orthologue of *HWS* and show the complexity of interaction between *EP3* and *OsFBK1* genes at different developmental stages of the plant. qRT‐PCR expression analyses and studies of *EP3‐GFP* and *OsFBK1‐RFP* promoter reporter lines demonstrate that although *EP3* and *OsFBK1* expression can be detected in the same tissues some cells exclusively express *EP3* or *OsFBK1* whilst others co‐express both genes. Loss, reduction or gain‐of‐function lines for *EP3* and *OsFBK1*, show that *EP3* and *OsFBK1* affect plant architecture, organ size, floral organ number and size, floral morphology, pollen viability, grain size and weight. We have identified the putative orthologue genes of the rice microRNA pathway for *ORYZA SATIVA DAWDLE (OsDDL*) and *ORYZA SATIVA SERRATE* (*OsSE*), and demonstrated that *EP3* and *OsFBK1* affect their transcript levels as well as those of *CROWN ROOT DEFECT 1*/*ORYZA SATIVA Exportin‐5 HASTY* (*CRD1/OsHST*), *ORYZA SATIVA DICER‐LIKE 1* (*OsDCL*) and *ORYZA SATIVA WEAVY LEAF1* (*OsWAF1*). We show that *EP3* affects OsPri‐MIR164, *OsNAM1* and *OsNAC1* transcript levels. *OsNAC1* transcripts are modified by *OsFBK1*, suggesting two independent regulatory pathways, one via *EP3* and *OsMIR164* and the other via *OsFBK1*. Our data propose that *EP3* and *OsFBK1* conjointly play similar roles in rice to how *HWS* does in Arabidopsis.

## Introduction

Ubiquitin protein degradation is a regulatory mechanism that affects many cellular processes including cell cycle, embryogenesis, development, floral formation, hormonal signalling, responses to stress and immunity in plants (Reed, 2003; Zhang *et al*., 2019). The level of key regulator proteins that modulate these processes is controlled by the 26S proteasome, which recruits E1, E2 and E3 enzymes to attach ubiquitin to proteins fated for degradation (Collins and Goldberg, 2017). The E3 ligase enzyme confers the required specificity by binding to the target substrate and the activated ubiquitin E2 complex, which is polyubiquitinated and then targeted for degradation by the 26S proteasome (Sharma *et al*., 2016). In rice, 1,332 types of E3 ligases have been reported (Du *et al*., 2009). The SCF complex, a kind of E3 ligase, is composed of four subunits: S‐phase‐kinase‐associated protein‐1 (Skp1), Cullin (Cul1), RING‐finger protein (Rbx1/Roc1) and F‐box protein (Yu and Matouschek, 2017), The F‐box protein confers the specificity for recognition of correct targets for degradation (Petroski and Deshaies, 2005). In rice, 687 F‐box proteins have been identified and shown to have diverse expression patterns, suggesting a range of roles during plant growth and development in response to internal and external signals (Jain *et al*., 2007).

The *ERECT PANICLE 3/LARGER PANICLE* (*EP3/ LP*, *Os02g15950*) and *ORYZA SATIVA F‐BOX KELCH 1* (*OsFBK1/ Os01g47050*) are two of the 687 F‐box reported genes from rice. EP3 and OsFBK1 proteins share in excess 50% amino acid sequence homology to the Arabidopsis HWS F‐box protein (Borah and Khurana, 2018; Yu *et al*., 2015).

The *EP3* gene was first identified during the description of the *erecta panicle 3* (*ep3*) mutant. The characteristic upright panicle phenotype *of ep3* is due to an increase in small vascular bundles number and by their wider parenchymal tissues in the peduncles. Seed production is reduced in *ep3* (Piao *et al*., 2009). In 2011, Li *et al* described the characterization of two allelic mutants: *larger panicle‐1* (*lp1*) and *2* (*lp2*), which have a robust plant architecture, and bigger panicles resulting in higher yields. The mapping of these mutants indicated that the affected gene was *Os02g15950* and these are allelic mutants to *ep3*. The mutant *ep3* has a diminished leaf photosynthetic capacity and stomatal conductance due to reduction in size of stomatal guard cells (Yu *et al*., 2015). In 2007, Jain *et al*, identified *OsFBK1* during a genome‐wide analysis of F‐box proteins in rice, and they demonstrated that *OsFBK1* expression is higher during panicle development and during the development of roots of 7‐day‐old seedlings. *OsFBK1* plays a role in regulating responses to drought (Borah *et al*., 2017). OsFBK1 targets two proteins for degradation: ORYZA SATIVA CINNAMOYL‐COA REDUCTASE (OsCCR14) and OsATL53. OsCCR14 is a protein involved in the synthesis of lignin in roots and anthers of rice (Borah and Khurana, 2018), and OsATL53, is an E3 ligase protein that interacts with OsCCR14 and affects its enzymatic activity (Borah *et al*., 2021).

*HAWAIIAN SKIRT* (*HWS*), an F‐box gene from Arabidopsis, is a regulator of plant growth, boundary formation and flower development (González‐Carranza *et al*., 2007). Loss‐ and gain‐of‐function lines from this gene are pleiotropic. Loss‐of‐function lines show floral sepal fusion (González‐Carranza *et al*., 2007). This is a phenotype shared with the double mutant of *cuc1/cuc2 [CUP‐SHAPED COTYLEDON1 (CUC1)* and *2 (CUC2)]* and *Pro35:164B* ectopic lines for the microRNA gene *MIR164B* (Aida *et al*., 1997; Laufs *et al*., 2004; Mallory *et al*., 2004; Mallory and Vaucheret, 2006). *HWS* loss‐of‐function plants are bigger, have longer roots and produce bigger seeds (González‐Carranza *et al*., 2007). *HWS* affects cell proliferation and controls size and floral organ number by indirectly regulating the accumulation of the transcripts of *CUC1* and *CUC2* (Gonzalez‐Carranza *et al*., 2017). *HWS* is involved in the microRNA pathway (Lang *et al*., 2018; Zhang *et al*., 2017). We have demonstrated that *EP3* is a functional orthologue of *HWS* (Yu *et al*., 2015). We hypothesize that *OsFBK1* is a functional orthologue of *HWS* and that *EP3* and *OsFBK1* have similar functioning mechanisms to the Arabidopsis *HWS* gene in the miRNA pathway, additional to these reported by the Khurana’s group.

Here we show that *OsFBK1* is a second functional orthologue of the Arabidopsis *HWS* gene. With the aid of fluorescent reporter genes and using qRT‐PCR, we describe the patterns of expression of *EP3* and *OsFBK1* at transcript level and at whole plant and organ level. We report the effect that loss‐ or gain‐of‐function lines for these genes has on plant architecture, flower and grain development and yield. We show that *EP3* affects transcript levels of OsPri‐MIR164, *OsNAM1 and OsNAC1. OsFBK1* alters *OsNAC1*, but not OsPri‐MIR164 transcript levels, suggesting that *OsNAC1* may be indirectly affected by *EP3* via *OsMIR164*, but also by an independent regulatory pathway where *OsFBK1* may be involved. We show that transcripts of the rice *CRD1/OsHST* (Zhu, *et al*., 2019), *OsDCL* and *OsWAF1* reported microRNA biogenesis pathway genes are altered by *EP3 and OsFBK1*. We report the identification of putative rice orthologues of *DDL*, *SE* and Os*HASTY* (named as CRD1 by Zhu *et al*., 2019). The transcript levels of these genes are altered by *EP3* and *OsFBK1* and likely to be via protein degradation of a target yet to be identified.

## Results and discussion

### *OsFBK1* is a rice functional orthologue of the *Arabidopsis HWS* gene

*EP3* is a functional orthologue of *HWS* (Yu *et al*., 2015). In 2007, Jain *et al* reported 687 potential F‐box proteins in rice. Of these, the closest homologue to *EP3* is *OsFBK1*. OsFBK1 shares 57% and 54% sequence homology with the rice EP3 and Arabidopsis HWS proteins respectively. To determine if *OsFBK1* is also a functional orthologue of *HWS*, the predicted *OsFBK1* cDNA coding region (1.236 Kb) was cloned using cDNA from the Nipponbare ecotype and sub‐cloned into the vector pBI101.2:*HWS_pro_* (González‐Carranza *et al*., 2007). Columbia‐0 (Col‐0) WT and *hws‐1* mutant plants were transformed and flower phenotypic comparisons between transgenic plants and controls were carried out. Flowers of the *hws‐1* plants failed to shed their sepals, which remained fused throughout pod development and senescence. Flowers from transformed plants with pBI101.2:*HWS_pro_:OsFBK1* in both the Col‐0 (Figure S1C,G) and *hws‐1* (Figure S1D,H) backgrounds resembled those of the Col‐0 WT plants, sepal fusion was absent (Figure S1B,F) and floral organ shedding was restored to similar levels to the Col‐0 WT (Figure S1A,E). Our results show that *OsFBK1* is a functional rice orthologue of *HWS*, suggesting that the role of *OsFBK1* during rice development may be similar to that of *HWS* in Arabidopsis, and that *HWS* has undergone a process of duplication in rice.

### *EP3* and *OsFBK1* transcripts accumulate in the same tissues

**Figure 1:**
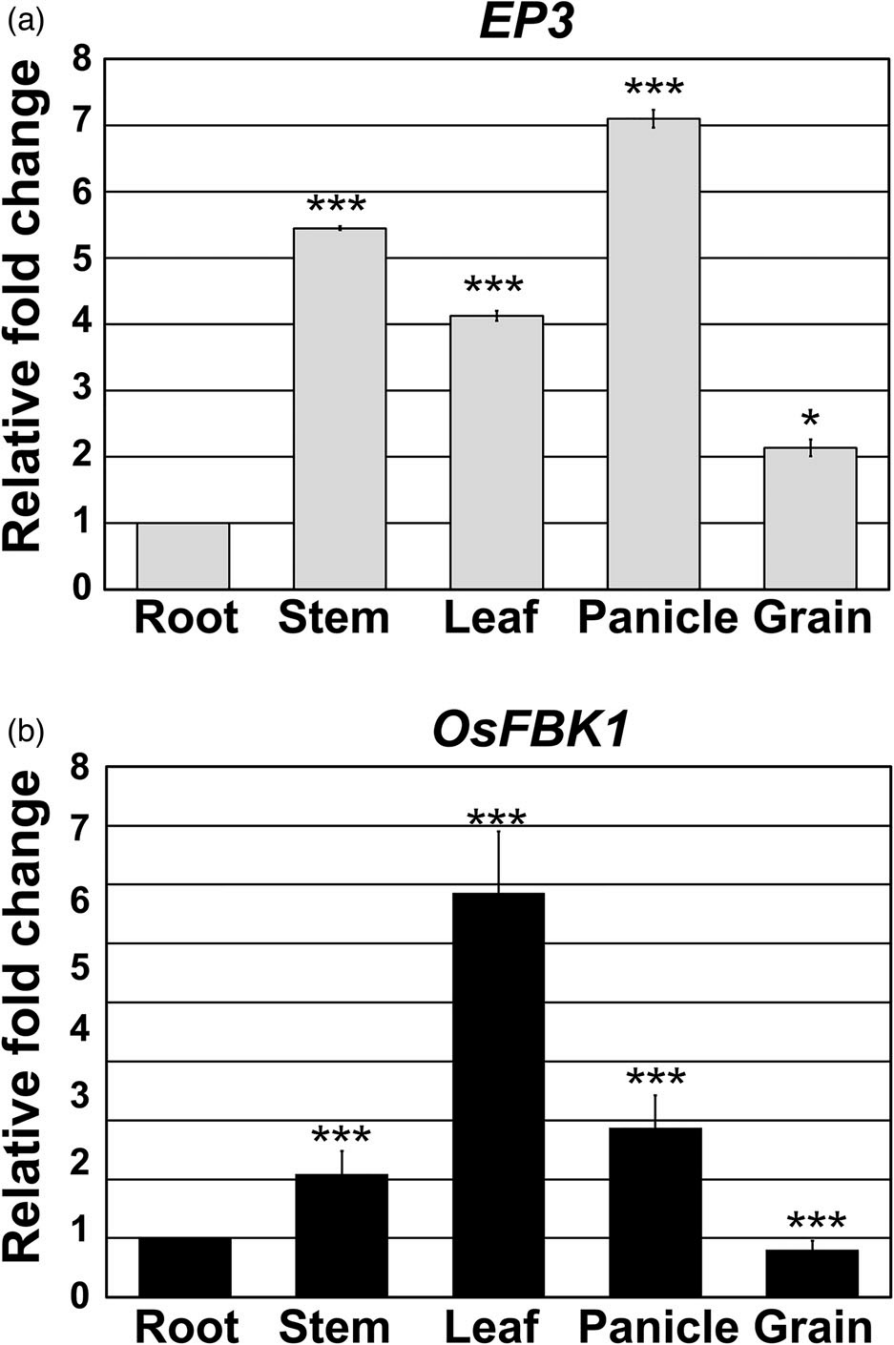
Differential expression of (A) *EP3* and (B) *OsFBK1* transcripts quantified by qRT‐PCR. Tissues analysed include: root and stem (7‐day‐old plants), leaf (number 6), panicle (15–20 cm) and grain (milk stage). Means ± SD are shown. Data are displayed as the ratio of expression to roots. Three biological replicates per tissue were used during these analyses. Single and triple asterisks show significant differences determined by Student’s *t*‐tests at *P < 0.05 and ***P < 0.0001 respectively.

To establish if the expression pattern of *EP3* is similar to that of *OsFBK1*, we analysed *EP3* and *OsFBK1* transcript levels in rice tissues using real‐time PCR (qRT‐PCR). Results revealed differences in transcript accumulation relative to the expression in roots. A statistically significant *EP3* over‐expression was observed in panicle (10–15 cm long), followed by stem, leaf and grain; with increases of 7, 5.5, 4‐ and 2‐ fold change respectively (Figure 1A). Relative to the expression in roots, a statistically significant *OsFBK1* over‐expression was observed in leaf, followed by panicle and stem with increases of 6‐, 3‐ and 2‐fold changes respectively. *OsFBK1* expression in grains was lower than that observed in roots (Figure 1B). These findings show that *EP3* and *OsFBK1* are most highly expressed in stems, leaves and panicles compared to roots and *OsFBK1* expression in grains is lowest in milking stage grains.

Our results confirm the expression reported for *EP3* by Piao *et al*. (2009) and Li *et al*. (2011), but differ from those reported for *OsFBK1* by Borah and Khurana (2018). It is possible that the discrepancies observed in our expression analyses and those of Borah and Khurana (2018) are due to the differences in growing conditions or due to the use of different varieties. In our studies, we used Nipponbare *japonica*, and Borah and Khurana (2018) used Pusa Basmati1 *indica*. These subspecies exhibit different genetics, morphologies and physiologies (Yang *et al*., 2014).

### GFP and RFP reporter analyses reveal expression or co‐expression of *EP3* and *OsFBK1*

**Figure 2:**
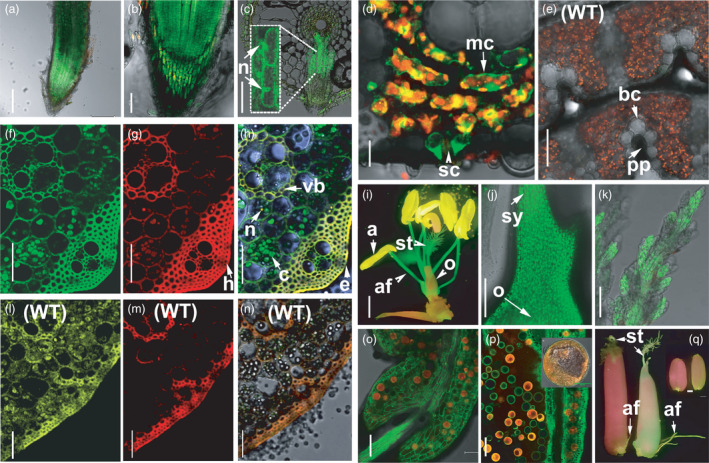
Co‐expression analyses from *EP3_pro_:GFP* and *OsBFK1_pro_:RFP* in T0 double transgenic reporter lines. Confocal images of (A‐C) roots, (D‐E) coiled 6th leaf, (F‐H and L‐N) stem, (I‐K) female flower, (O‐P) anther and pollen and (Q) seeds. (A) primary root, (B) root cap, and (C) close up section of emerging lateral roots, magnified cells show nuclear localization of *EP3_pro_:GFP*, (D) transversal cross sections of leaves in T0 reporter lines and (E) in WT. (F‐H) GFP, RFP and merged images of transversal cross sections of stem in T0 reporter lines and (L‐N) in WT plants. (I) Dissected flower. (J and K) Close up of style and of stigma respectively. (O) anther‐stamen junction. (P) Pollen and close up of pollen. (Q) Filling and mature grains. Developmental stages: roots are from 7‐day‐old seedling plants, stems and leaves from 45‐day‐old plants, flowers from 60‐day‐old plants and milky stage and dry grains and from 65‐ and 70‐day‐old plants respectively. n: nucleus, bc: bulliform cells, pp: proto phloem, mc: mesophyll cells, sc: stomata complex, h: hypodermis, vb: vascular bundles, c: chloroplasts, e: epidermis, a: anther, af: anther filament, st: stigma, o: ovary, sy: style. Scale Bars: A‐C, J and P = 100 µm, D‐E = 50 µm, F‐H and L‐N = 45 µm, I and Q = 1 mm and O = 20 µm.

To determine the cellular expression of *EP3* and *OsFBK1* during plant development single and double reporter transgenic lines in Nip background were generated. Genomic regions of 2.686 Kb and 2.739 Kb containing the promoters and 5’UTRs of *EP3* and *OsFBK1* were fused to GFP and RFP reporter genes respectively. A double transgenic line was generated by transforming calli with *EP3_pro_::GFP* and *OsFBK1_pro_::RFP* constructs. Expression was examined in T0 double transgenic lines using fluorescence or confocal microscopy. Results showed expression of *EP3_pro_::GFP* and *OsFBK1_pro_::RFP* in cells of roots, stems, leaves, flowers and seeds (Figure 2). *EP3_pro_::GFP* was highly expressed in cells of primary and secondary roots, meristematic zones cells, root cap cells and epidermis, and its expression was observed both in cytoplasm and in nucleus. The expression of *OsFBK1_pro_::RFP* was detected at low levels in the nucleus of meristematic zone cells, epidermis and root cap cells (Figure 2A–C). Transversal sections of leaf number six showed expression of *EP3_pro_::GFP* in cytoplasm of proto‐phloem, stomata and bulliform cells; and in nucleus of proto‐phloem and bundle sheath cells. Co‐expression of *EP3_pro_::GFP* and *OsFBK1_pro_::RFP* seen as orange colour compared with the red fluorescence produced by chlorophyll, was observed in mesophyll cells (Figure 2D,E). In stems, co‐expression of *EP3_pro_::GFP* and *OsFBK1_pro_::RFP* was observed in hypodermis, epidermis, nucleus and bundle sheaths cells (Figure 2F–H). Images from WT stems are included for comparison (Figure 2L–N). Confocal floral analyses revealed that *EP3_pro_::GFP* was expressed in both the nucleus and cytoplasm of anther filaments, stamen, styles and stigma. *OsFBK1_pro_::RFP* was present in tapetum cells and co‐expression of *EP3_pro_::GFP* and *OsFBK1_pro_::RFP* was observed in anthers, in the joining tissue between the anther and the anther filament, in pollen grains and developing ovaries (Figure 2I–K, O–Q). Fifty percent of pollen grains showed *RFP* expression confirming a single copy of *mRFP1* gene insertion in the primary transgenic plants (Figure 2P). *OsFBK1_pro_::RFP* expression was absent in early stages of pollen development, but present in mature pollen. These results confirm the *EP3* and *OsFBK1* expression observed in tissues analysed by qRT‐PCR and reveal cellular and subcellular localization of expression or co‐expression of *EP3* and *OsFBK1*. These data demonstrate that *EP3* and *OsFBK1* are co‐expressed in some tissues. Some cells exclusively express either *EP3* or *OsFBK1* suggesting these genes may act independently in some cells and interdependently in others. Our results show that *EP3* and *OsFBK1* genes are expressed both in the nucleus and in the cytoplasm of cells from meristematic zones, roots, stems, leaves, flowers and seeds. We have been unable to generate functional EP3 and OsFBK1 translational fusions lines containing GFP or RFP tags proteins. It is likely the GFP and RFP tags interfere with the functionality of these F‐box proteins *in planta*.

### Loss, reduction or gain‐of‐function lines for *EP3* and *OsFBK1* are functional

In the absence of HWS expression, Arabidopsis plants are more robust and produce bigger plant organs, including rosettes, roots and seeds (Gonzalez‐Carranza *et al*., 2007). We hypothesized that the lack of expression of *EP3* and *OsFBK1* would have similar effects in rice and the opposite would be true when these genes are over‐expressed. To test this hypothesis, to determine the effect of *OsFBK1* in rice development, and to understand the synergism between *EP3* and *OsFBK1* in shaping plant architecture, we generated and studied *EP3* and *OsFBK1* loss/reduced and gain‐of‐function rice plants. Homozygous plants from *ep3* (Piao *et al*., 2009), *osfbk1* ^RNAi^, *EP3^OE^*, *OsFBK1* ^OE^ and *ep3/osfbk1* ^RNAi^ were created, identified and analysed in these experiments. To confirm *EP3* and *OsFBK1* transcript absence or accumulation in loss‐ and gain‐of‐function lines generated, we performed qRT‐PCR in 10‐15 day old panicle tissues from three F2 plants from our *ep3* (Piao *et al*., 2009), *osfbk1* ^RNAi^, *EP3^OE^*, *OsFBK1* ^OE^ and *ep3/osfbk1* ^RNAi^ lines each. The same plants used to perform these analyses were also used in the phenotypic analyses described later. Expression patterns of *EP3* and *OsFBK1* for these lines are included in Figure 5 to facilitate comparison with expression patterns of putative genes influenced by *EP3* and *OsFBK1* and described later in the paper. Significant 38‐ and 2.3‐ fold increases were observed in *EP3* transcript levels of *EP3^OE^* and *osfbk1* ^RNAi^ lines respectively (Figure 5A). The *ep3* and *ep3/osfbk1* ^RNAi^ lines showed a reduction in *EP3* transcript but not a complete reduction. The *ep3* mutation introduces an earlier termination codon likely to produce a truncated protein of 266 aa. The primers used in the qRT‐PCR analyses are located before the stop codon introduced earlier. It is likely these primers are detecting transcripts of the truncated protein showing in our analyses as a reduced expression of *EP3*. We used three biological replicates of these experiments, and this may explain why a high SD is observed in *ep3* and *ep3/osfbk1* ^RNAi^ lines. Transcript levels of *OsFBK1* were increased in *ep3* (*P* < 0.05) and *OsFBK1* ^OE^ (*P* < 0.001) by 3‐ and 27‐fold respectively. Significant reductions of *OsFBK1* transcript were observed in *EP3^OE^*, *osfbk1* ^RNAi^ and *ep3/osfbk1* ^RNAi^ of 0.3, 0.6 and 0.75 respectively (Figure 5B). These results confirm that our ectopic lines are over‐expressing *EP3* and *OsFBK1* transcripts. Our *osfbk1* ^RNAi^ is likely to be a knockdown with reduction of *OsFBK1* transcript of about 2/3 compared to WT Nip. *OsFBK1* transcript is reduced in *ep3/osfbk1* ^RNAi^.

We attempted to generate a double knockout from *EP3* and *OsFBK1*. Although we were unable to completely silence *EP3* and *OsFBK1* expression, we reduced *OsFBK1* expression by about two‐thirds compared to the wild type (Figure 5A,B). Analyses of our double *EP3* KO and *OsFBK1* knockdown lines show that the flowers of these plants display floral fusion and abnormal growths at the top of the stigma as do the single knockdown lines (Figure 4). Floral fusion has been observed in the *hws‐1* mutant from Arabidopsis (Gonzalez‐Carranza, *et al*., 2007), suggesting that the function of *HWS* and *EP3* and *OsFBK1* during flower development may be conserved in Arabidopsis and rice.

### *EP3* and *OsFBK1* alter plant architecture

**Figure 3:**
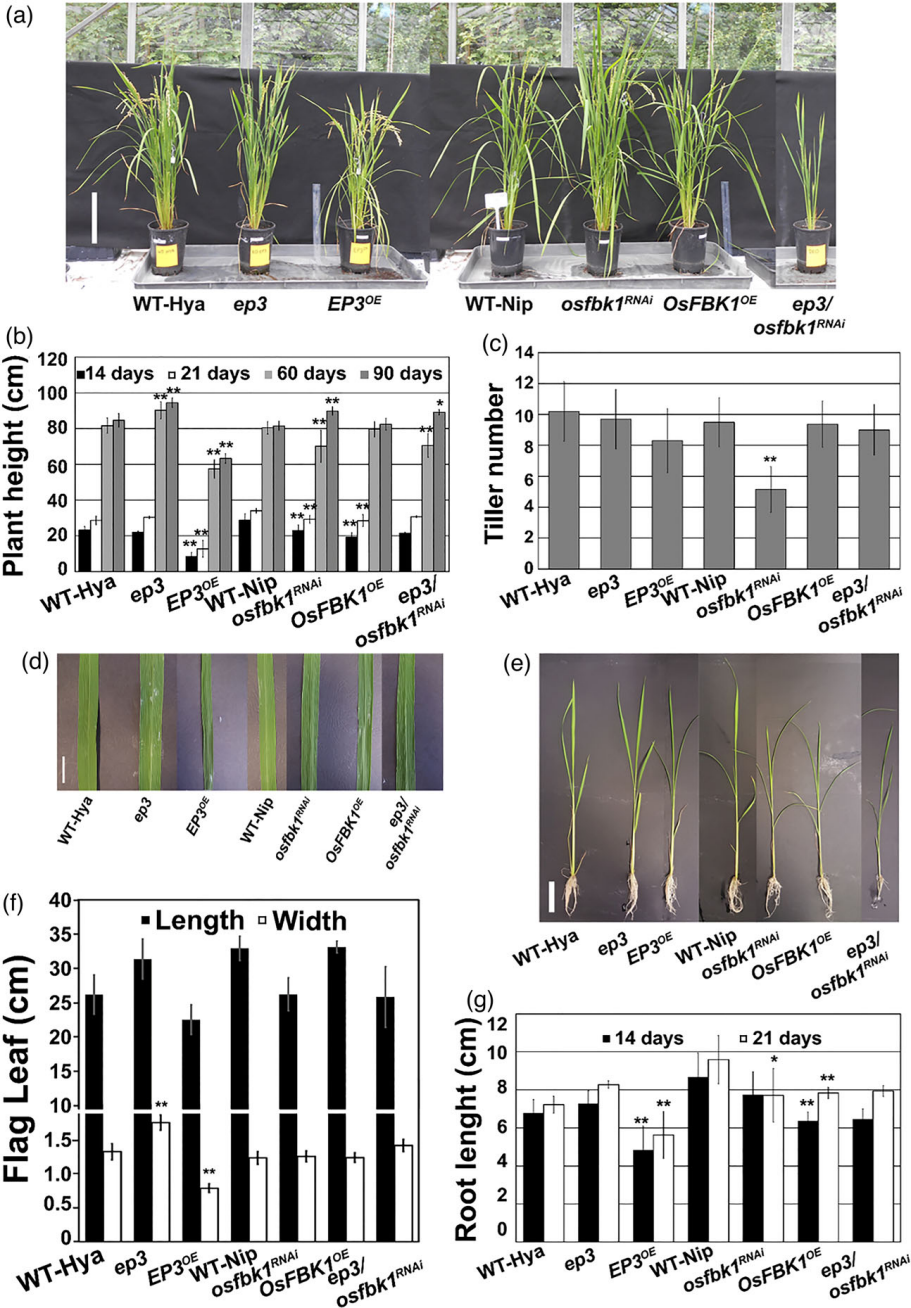
*EP3* and *OsFBK1* regulate plant growth. (A) 90‐day‐old plants. (B) Plant height at 14, 21, 60 and 90 days. (C) Tiller number of 60‐day‐old plants. (D) Differences in leaf width. (E) 21‐day‐old plants showing differences in root growth. (F) Differences in Flag leaf dimensions. (G). Differences in root growth in 14‐ and 21‐day‐old plants. Plants in all pictures and graphs include: WT‐Hya, *ep3*, *EP3^OE^*, WT‐Nip, *osfbk1^RNAi^*, *OsFBK1^OE^* and ep3/*osfbk1^RNAi^*. The *ep3*, *EP3^OE^* and ep3/*osfbk1^RNAi^* lines are in Hya background, while *osfbk1^RNAi^*, *OsFBK1^OE^* are in Nip background. Scale bars: (A) = 15 cm, (D) = 2cm and (E) = 5 cm. Means ± SD are shown in (B, C, F and G). n values: (B and G) *n = 51*, and (C and F) *n = 56*, (G). Single and double asterisks show significant differences determined by Student’s *t*‐tests at *P* < 0.05 and *P* < 0.01 respectively.

Morphological studies of the *ep3* (Piao, *et al*., 2009), *osfbk1* ^RNAi^, *EP3^OE^*, *OsFBK1* ^OE^ and *ep3/osfbk1* ^RNAi^ lines were performed throughout the development of plants grown in soil except for root length analyses that were performed in plants growing in hydroponics (Figure 3). Plant height of homozygous plants for *EP3* and *OsFBK1* loss/reduced and gain‐of‐function was analysed at 14, 21, 60 and 90 days (Figure 3A,B). The *ep3* mutant did not show differences at 14 or 21 days, but 60‐ and 90‐day‐old plants were taller than WT‐Hya by about 10 cm. *EP3^OE^* plants were shorter than WT‐Hya by 10–20 cm throughout development. The *osfbk1* ^RNAi^ line showed shorter plants than the WT‐Nip at 14, 21 and 60 days. At 90 days, these plants were taller than WT‐Nip by about 10 cm. *OsFBK1* ^OE^ plants were shorter than WT‐Nip plants at 14 and 21 days, but not different at 60 and 90 days compared to WT‐Nip. The *ep3*/*osfbk1* ^RNAi^ line, which was generated by transforming the *ep3* mutant (Hya background) showed shorter plants at 60 days and higher plants at 90 days compared to WT‐Hya (Figure 3A,B). Tiller number of 60‐day‐old plants from all homozygous lines was analysed. Only *osfbk1* ^RNAi^ plants showed reduced numbers of tillers—about half—compared to WT‐Nip (Figure 3C). Leaf length and width of fully expanded flag leaves from 90‐day‐old plants from all homozygous lines were measured. The *ep3* and *EP3^OE^* flag leaves were wider and narrower respectively, about 1 cm each, compared to WT‐Hya (Figure 3D,F). Homozygous plants from all lines were grown in hydroponics and the length of their roots was measured at 14 and 21 days. Shorter roots were observed at 21 days in *EP3^OE^* compared WT‐Hya and in *osfbk1* ^RNAi^ and *OsFBK1* ^OE^ compared to WT‐Nip. No significant differences were observed in other lines or in 14‐day old plants (Figure 3E,G).

Our results show that *ep3* plants are taller and have wider leaves than the *EP3* over‐expressing plants. Plants with reduced transcript levels of *OsFBK1* are smaller during the initial developmental stages and taller after 90 days. They also have reduced tiller numbers. Down‐regulation of *EP3* and *OsFBK1* also produces smaller plants that recover at 90 days. *EP3* and *OsFBK1* over‐expression produces smaller plants, with *OsFBK1* over‐expression plants recovering as development progresses. Our results suggest a complex interaction between *EP3* and *OsFBK1* affecting plant architecture. We cannot rule out that these differences are due to the differences in background of the mutants used. This data reveals a time‐related effect of *EP3* and *OsFBK1* during rice development.

### Panicle, grain development and yield are affected by *EP3* and *OsFBK1*

To determine if the changes in plant architecture observed in our lines are associated with seed production and yield, we analysed panicle, grains and yield from five plants from each line generated and their controls. Consistent with previous reports (Piao *et al*., 2009; Yu *et al*., 2015) mature stage panicles of *ep3* mutant were significantly shorter than those of WT‐Hya. Panicles of *OsFBK1* ^OE^ were also significantly shorter than WT‐Nip. *EP3^OE^*, *osfbk1* ^RNAi^ and *ep3/osfbk1* ^RNAi^ were not different to WT controls. *EP3^OE^* and *OsFBK1* ^OE^ lines showed significantly less grains per panicle compared to WT controls (22 and 17 less grains respectively). No difference in grains per panicle was observed in *ep3*, *osfbk1* ^RNAi^ and *ep3/osfbk1* ^RNAi^ lines. Significant reductions in filled grain number per panicle was observed in all lines studied compared to WT controls. The *ep3/osfbk1* ^RNAi^ and *EP3^OE^* produced on average 4 and 10 filled grains compared to 44 grains produced by WT (Table 1A, Figure S2A).

**Table 1:** Effect of *EP3* and *OsFBK1* on (A) panicle and (B) grain development

(A)			
Genotype	Panicle		
	Length (mm) (change vs WT)	Total grain/panicle (change vs WT)	Filled grain/panicle (change vs WT)
WT‐Hya	16.2 ± 1.3	56.0 ± 6.3	44.0 ± 7.9
*ep3*	12.6 ± 0.9*	55.8 ± 4.4	33.0 ± 5.5*
*EP3^OE^*	13.9 ± 1.1	34.2 ± 7.9**	9.6 ± 2.0**
*ep3/osfbk1*	15.5 ± 2.7	43.8 ± 11.6	3.6 ± 1.8**
WT‐Nip	19.0 ± 1.0	59.2 ± 6.8	41.0 ± 10.0
*osfbk1^RNAi^*	16.5 ± 2.2	45.6 ± 3.7	21.8 ± 3.4**
*OsFBK1^OE^*	13.2 ± 0.3**	41.8 ± 4.0**	24.2 ± 5.5**

(B)				
Genotype	Grain			
	Length (mm) (change vs WT)	Width (mm) (change vs WT)	Area (mm^2^) (change vs WT)	Weight of 100 dry grains (g) (change vs WT)
WT‐Hya	6.84 ± 0.47	2.94 ± 0.35	19.17 ± 2.54	2.341 ± 0.05
*ep3*	6.16 ± 0.57**	3.19 ± 0.45**	15.10 ± 4.14**	2.417 ± 0.09
*EP3^OE^*	5.72 ± 0.57**	2.07 ± 0.39**	10.10 ± 1.89**	1.574 ± 0.03**
*ep3/osfbk1*	4.83 ± 0.45**	2.16 ± 0.37**	10.88 ± 1.99**	1.987 ± 0.06**
WT‐Nip	6.61 ± 0.49	2.52 ± 0.41	16.33 ± 2.89	2.530 ± 0.08
*osfbk1^RNAi^*	6.82 ± 0.39**	2.61 ± 0.35	17.98 ± 2.33**	2.187 ± 0.03**
*OsFBK1^OE^*	8.43 ± 0.61**	3.22 ± 0.42**	21.79 ± 2.93**	2.731 ± 0.07*

Data show average values followed by ± SD. N values: (A) *n* = 35, (B) *n* = 700. The *ep3*, *EP3^OE^* and *ep3*/*osfbk1^RNAi^* lines are in Hya background, while *osfbk1^RNAi^*, *OsFBK1^OE^* are in Nip background. * and ** show significant differences at *P* < 0.05 and *P* < 0.01 respectively, determined by Student’s t‐test (A) and regression analysis (B).

Previous analysis of Arabidopsis seeds indicated that in the absence of a functional *HWS* gene, seeds are bigger. The opposite is true in gain of function mutants (González‐Carranza *et al*., 2007). To determine if *EP3* and *OsFBK1* alter rice grain size in a similar manner we measured the length, width, area and weight of 100 grains from mature panicles of each genotype. Grains of *ep3*, *EP3^OE^* and *ep3/osfbk1* ^RNAi^ were shorter than WT‐Hya. In contrast, grains of *osfbk1* ^RNAi^ and *OsFBK1* ^OE^ were longer than WT‐Nip. Grains of *ep3* were wider than WT‐Hya, while grains of *EP3^OE^* and *ep3/osfbk1* ^RNAi^ were narrower. Grains of *osfbk1^OE^* were wider than WT‐Nip. The width of grains from the *OsFBK1^RNAi^* line were no different to WT Nip. Grain areas of *ep3*, *EP3^OE^* and *ep3/osfbk1* ^RNAi^ were smaller than WT‐Hya, while grain areas of *osfbk1* ^RNAi^ and *OsFBK1* ^OE^ were bigger than WT‐Nip. Grains of *EP3^OE^* and *ep3/osfbk1* ^RNAi^ were lighter than WT‐Hya. Grains of *osfbk1* ^RNAi^ and *OsFBK1* ^OE^ were lighter or heavier than WT‐Nip respectively. The grains from *ep3* were not different to the control WT (Table 1B, Figure S2B). These data show that *EP3* and *OsFBK1* affect yield by influencing panicle architecture, grain size and weight. Our results show that both *EP3* and *OsFBK1* affect grain filling. The reduction of seed size in the *EP3^OE^* line is consistent with our findings in Arabidopsis, that is, when the *HWS* gene is ectopically expressed the seeds are smaller. Surprisingly, reducing the expression of *OsFBK1* results in bigger but lighter grains, while over‐expressing *OsFBK1* results in bigger and heavier grains. When the expression of both *EP3* and *OsFBK1* are reduced, grains are considerably smaller and lighter than when the expression of only one of the genes is non‐functional or down‐regulated, or when the *EP3* is over‐expressed. These results suggest that *EP3* and *OsFBK1* act antagonistically to regulate grain size and weight.

The functionality of F‐box proteins relies on their interaction with other proteins of the SCF complex through domains in their N terminus (Petroski and Deshaies, 2005), and in the substrate specificity conferred by their C‐terminus domain (Zhang *et al*., 2019). Li *et al*. (2011) identified two mutants of the *EP3* gene: the *lp1* and *lp2*, which are more robust, vigorous and productive compared with WT plants. The differences we observed in our *ep3* studies are possibly due to the position and nature of the mutations in each line. The *ep3* mutation is located in the middle of the gene, outside the F‐box domain, and introduces an earlier termination codon resulting in a truncated protein (Piao *et al*., 2009). The *lp1* mutation generates a protein of the same size as the WT, but with a Serine to Proline amino acid substitution at position 472. The *lp2* mutation generates a longer protein than the WT by 21 amino acids caused by deletion of two nucleotides and a frameshift (Li *et al*., 2011). It is possible that in the *ep3* lines, the interactions of the shorter EP3 peptide with the SCF complex and its target(s) are compromised, not allowing the protein to function properly. Detailed deletion analyses and amino‐acid residue specificity of EP3 and OsFBK1, outside of the scope of this study, would provide more information on the mechanisms regulating grain yield and plant architecture in rice.

### Flower organ number and morphology is altered by *EP3* and *OsFBK1*

**Figure 4:**
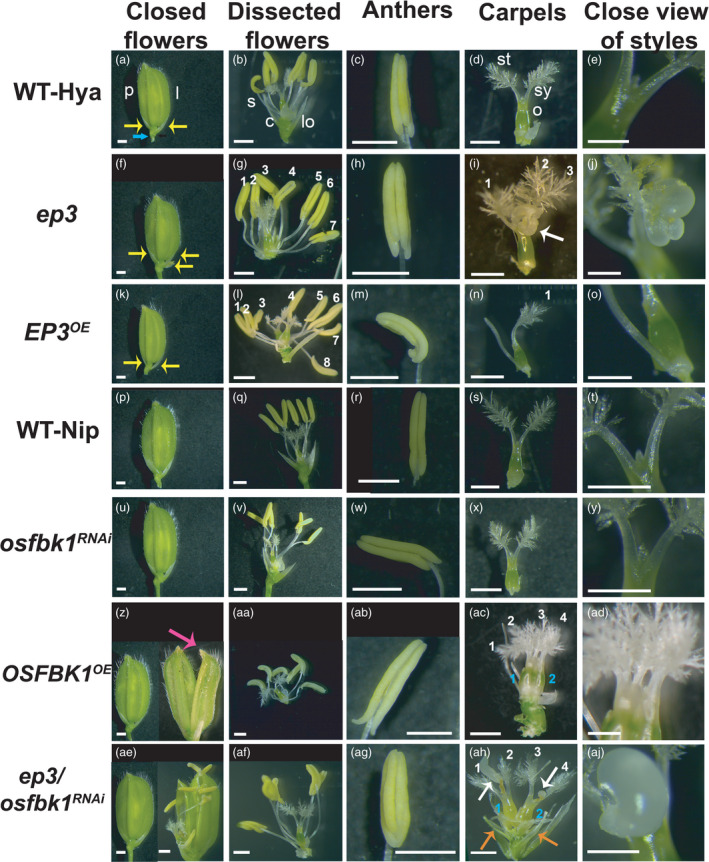
*EP3* and *OsFBK1* affect flower development and floral organ number and size. (A, F, K, P, U, Z, and AE) Closed flowers: just before anthesis showing palea (p) and lemma (l). (B, G, L, Q, V, AA and AF) dissected flowers without palea and lemma to show the inner floral whorls: lodicules (lo), stamens (s) and carpels (c). (C, H, M, R, W, AB and AG) anthers. (D, I, N, S, X, AC and AH). Carpels showing stigma (st), style (sy) and ovaries (o). (E, J, O, T, Y, AD and AJ) Close up view of the style and ovary junction. Phenotypes shown include: (A‐E) WT‐Hya, (F‐J) *ep3*, (K‐O) *EP3^OE^*, (P‐T) WT‐Nip, (U‐Y) *osfbk1^RNAi^*, (Z‐AD) *OsFBK1^OE^* and (AE‐AJ) ep3/*osfbk1^RNAi^*, note the double flower in the right side of the single flower in AE. The *ep3*, *EP3^OE^* and ep3/*osfbk1^RNAi^* lines are in Hya background, while *osfbk1^RNAi^*, *OsFBK1^OE^* are in Nip background. Yellow, blue, pink, white and orange arrows show sterile glumes, rudimentary glumes, extra glume, abnormal growths at the top of the ovary and velum‐like structures respectively. White numbers in G and L show anther number, white numbers in I, N, AC and AH show stigma number. Blue numbers in AC and AH show ovary number. Scale bars: 1mm, except for (E, J, O, T, Y, AD and AJ) where the scale is 500 µm.

Our studies in Arabidopsis have shown that *HWS* affects size and organ flower number by modulating *CUC1* and *CUC2* transcript levels (Gonzalez‐Carranza *et al*., 2017). *HWS* alters sepal boundary formation in Arabidopsis flowers (Gonzalez‐Carranza *et al*., 2007). We hypothesize that *EP3* and *OsFBK1* play similar roles in rice. To determine if *EP3* and *OsFBK1* also regulate size and floral organ number in rice and to analyse if the reduction of grain filling is due to changes in floral morphology and pollen viability we studied fully mature flowers from our *ep3* (Piao *et al*., 2009), *osfbk1* ^RNAi^, *EP3^OE^*, *OsFBK1* ^OE^ and *ep3/osfbk1* ^RNAi^ lines. The number of glumes was increased in the *OsFBK1* ^OE^ and *ep3/osfbk1* ^RNAi^ with a significant increase compared to WT‐Nip and WT‐Hya controls respectively. The number of stamens was also increased in *ep3* and *OsFBK1* ^OE^ compared to WT‐Hya and WT‐Nip respectively. The number of stigmas increased in *ep3* and *ep3/osfbk1* ^RNAi^ compared to Wt‐Hya while the carpel number was increased in *OsFBK1* ^OE^ compared to WT‐Nip (Table 2A; Figure 4).

**Table 2:** Effect of *EP3* and *OsFBK1* on (A) floral organ number, (B) spikelet and stigma size and (C) anther size and pollen viability

(A)				
Genotype	Number of:			
	Glumes (palea + lemma)	Stamens	Stigmas	Carpels
WT‐Hya	2.00 ± 0.00	6.00 ± 0.00	2.00 ± 0.00	1.00 ± 0.00
*ep3*	2.05 ± 0.22	6.24 ± 0.65*	2.27 ± 0.45**	1.00 ± 0.00
*EP3^OE^*	2.00 ± 0.00	6.21 ± 0.51	1.89 ± 0.31	1.11 ± 0.31
*ep3/osfbk1*	2.19 ± 0.45**	6.02 ± 0.12	2.28 ± 0.45**	1.00 ± 0.00
WT‐Nip	2.00 ± 0.00	6.00 ± 0.00	2.00 ± 0.00	1.00 ± 0.00
*osfbk1^RNAi^*	2.00 ± 0.00	6.00 ± 0.00	2.00 ± 0.00	1.00 ± 0.00
*OsFBK1^OE^*	2.18 ± 0.39**	6.10 ± 0.37**	2.06 ± 0.25	1.18 ± 0.39*

(B)						
Genotype	Spikelet			Stigma		
	Length (mm)	Width (mm)	Area (mm^2^)	Length (mm)	Width (mm)	Area (mm^2^)
WT‐Hya	6.83 ± 0.09	3.16 ± 0.12	16.78 ± 0.54	1.16 ± 0.11	0.67 ± 0.15	0.66 ± 0.21
*ep3*	6.78 ± 0.09	3.69 ± 0.09**	18.72 ± 0.66**	1.25 ± 0.14	0.75 ± 0.16	1.16 ± 0.33**
*EP3^OE^*	6.01 ± 0.16**	2.67 ± 0.15**	12.49 ± 0.54**	1.05 ± 0.06	0.54 ± 0.08	0.47 ± 0.11
*ep3/osfbk1*	6.92 ± 0.17	3.84 ± 0.14**	20.33 ± 0.89**	1.54 ± 0.25**	0.82 ± 0.18	1.02 ± 0.28*
WT‐Nip	7.05 ± 0.19	3.44 ± 0.08	19.07 ± 0.56	1.42 ± 0.14	0.83 ± 0.06	0.87 ± 0.09
*osfbk1^RNAi^*	7.17 ± 0.14	3.31 ± 0.22*	18.49 ± 1.05	1.11 ± 0.13**	0.57 ± 0.13**	0.54 ± 0.12**
*OsFBK1^OE^*	7.08 ± 0.13	3.38 ± 0.15	18.12 ± 0.63**	1.43 ± 0.15	0.71 ± 0.11*	1.20 ± 0.38*

(C)				
Genotype	Anther			Pollen Viability
	Length (mm)	Width (mm)	Area (mm^2^)	Percentage
WT‐Hya	1.57 ± 0.11	0.36 ± 0.02	0.59 ± 0.04	93.2 ± 1.60
*ep3*	1.85 ± 0.19**	0.45 ± 0.02**	1.30 ± 0.11**	85.8 ± 1.70**
*EP3^OE^*	1.27 ± 0.20**	0.37 ± 0.04	0.46 ± 0.08**	70.9 ± 2.20**
*ep3/osfbk1*	1.82 ± 0.18*	0.45 ± 0.04**	0.65 ± 0.07	67.9 ± 0.46**
WT‐Nip	2.01 ± 0.09	0.40 ± 0.02	0.71 ± 0.05	92.6 ± 1.10
*osfbk1^RNAi^*	1.90 ± 0.18	0.40 ± 0.03	0.61 ± 0.08*	87.2 ± 0.59*
*OsFBK1^OE^*	2.19 ± 0.18*	0.47 ± 0.03**	0.80 ± 0.09*	82.4 ± 2.80**

Data show average values followed by ± SD. N values: *n* = 63; except for pollen viability where *n* = 21. The *ep3*, *EP3^OE^* and *ep3*/*osfbk1^RNAi^* lines are in Hya background, while *osfbk1^RNAi^*, *OsFBK1^OE^* are in Nip background. * and ** show significant differences determined by Student’s *t*‐tests at *P* < 0.05 and *P* < 0.01 respectively.

During flower dissections, we observed that the size of floral organs was affected in the lines studied. Spikelets of *EP3^OE^* were shorter, narrower and occupied less area than WT‐Hya. In *ep3* and *ep3/osfbk1* ^RNAi^ spikelets were wider and occupied a bigger area than WT‐Hya. In *osfbk1* ^RNAi^ these organs were narrower than WT‐Nip, and the area they occupied in *OsFBK1* ^OE^ was smaller than WT‐Nip (Table 2B). About 6% of *ep3 s*pikelets displayed an extra glume outside the lemma or palea while about 13% of the *ep3/osfbk1* ^RNAi^ *s*pikelets had two or more fused florets (Figure 4A,F,K,P,U,Z, AE).

The area occupied by stigmas of *ep3* was bigger than WT‐Hya. In contrast, stigmas of *osfbk1* ^RNAi^ were shorter, narrower and occupied less area than WT‐Nip. Stigmas of *OsFBK1* ^OE^ were narrower but occupied a bigger area to WT‐Nip. Stigmas of *ep3/osfbk1* ^RNAi^ were longer, and occupied a bigger area compared to WT‐Hya (Table 2B, Figure 4D,E,I,J,N,O,S, T,X,Y,AC,AD,AH,AJ). In WT flowers, carpels began to elongate and start their development after successful fertilization (Yhosida and Nagato, 2011). However, in about 12% and 11% of flowers of *OsFBK1* ^OE^ and *ep3/osfbk1* ^RNAi^ respectively, elongation of carpels was observed before the onset of anthesis. Around 16% of *OsFBK1* ^OE^ flowers contained vellum‐like organs inside the flowers (orange arrows, Figure 4AH). *EP3^OE^*, *OsFBK1* ^OE^ and *ep3/osfbk1* ^RNAi^ flowers presented double carpels in about 11% and 17% of the over‐expressing lines and the double *ep3/osfbk1* ^RNAi^ line respectively (double ovary only shown for *OsFBK1* ^OE^ and *ep3/osfbk1* ^RNAi^ Figure 4AC,AH).

Anthers of *ep3* were longer, wider and occupied a bigger area compared to WT‐Hya. *EP3^OE^* anthers were shorter and occupied a smaller area compared to WT‐Hya. The pollen viability of *ep3*, *ep3/osfbk1* ^RNAi^ and *EP3^OE^* was reduced compared to WT‐Hya. The area occupied by anthers of *osfbk1* ^RNAi^ was smaller than WT‐Nip. In contrast, anther length, width and area of *OsFBK1* ^OE^ were larger than WT‐Nip. *OsFBK1* ^OE^ and *EP3^OE^* pollen viability, was reduced. Anthers of *ep3/osfbk1* ^RNAi^ were longer and wider compared to WT‐Hya and the pollen viability of *ep3/osfbk1* ^RNAi^ was lower than WT‐Hya (Table 2C, Figure 4B,C,G,H,L,M,Q,R,V, W,AA,AB,AF,AG). Pollen viability is hormonally regulated (Acosta and Przybyl, 2019). Intersections between phytohormone responses and the miRNA pathway have been previously reported (Liu and Chen, 2009). Analysing hormonal homeostasis in all our transgenic lines will increase our understanding of these interactions.

During our dissection analyses, we discovered that about 24% and 26% of the *ep3* and *ep3/osfbk1* ^RNAi^ flowers respectively presented an uncharacteristic growth at the top of the styles (Figure 4I,J,AH,AJ). Additionally, chimeric organs were observed in *ep3/osfbk1* ^RNAi^ flowers (white arrows, Figure 4AH). Ectopic anthers originating from the lodicule were also present in about 2% of flowers. Detailed anatomical analyses of these growths will allow us to determine the type of cells contained in them. Down‐regulation of *EP3* results in an increased number of stamens and stigmas, as well as bigger floral organs. Over‐expressing *EP3* does not significantly change floral organ number but results in smaller floral organs. Down‐regulation of *OsFBK1* does not alter floral organ number but results in smaller spikelets and bigger stigmas and anthers. Over‐expressing *OsFBK1* increases floral organ numbers and anther sizes but reduces spikelet sizes (Table 1A).

Our gain‐ and loss‐of‐function *EP3* and *OsFBK1* lines show increased numbers of glumes, stamens, stigmas and carpels, differences in floral organ sizes and reduction of pollen viability. These findings suggest that *EP3* and *OsFBK1* may influence or regulate the expression of floral homeotic genes from the ABCDE model in rice (Sugiyama *et al*., 2019; Yoshida and Nagato, 2011). Analyses of the expression of these genes and the generation of double KO lines would support this hypothesis. Careful analyses of flowers showed the presence of a tumour‐like growth at the top of the styles as well as the presence of chimeric organs in some floral organs highlighting a possible a role for *EP3* and *OsFBK1* in floral meristem regulation. Detailed anatomical, genetic and proteomic analyses of these plants will allow a greater understanding of the role of *EP3* and *OsFBK1* in flower development and meristem regulation.

Our anther analyses for *OsFBK1* are different to those reported by Borah and Khurana (2018). Their *OsFBK1^OE^* lines have shorter anthers while they are significantly longer in their *OsFBK1^KD^* lines. Our *OsFBK1^OE^* and *OsFBK1^RNAi^* lines showed larger and shorter anthers respectively. These differences may be due to the genotypes chosen, the cloning vectors used, the genomic areas selected to generate these lines, the areas in the genome where the insertions took place, and/or the environmental conditions used to grow the plants. Borah and Khurana (2018) used an *indica* (Pusa Basmati1) variety while we used a *japonica* (Nip). The vectors used by Borah and Khurana, 2018 are pB4NU (*OsFBK1^OE^*) and pANDA (*OsFBK1^KD^*), while we used pBRACT214 (*OsFBK1^OE^*) and pBRACT207 (*OsFBK1^RNAi^*) vectors. Both *OsFBK1^OE^* were created using a 1.236 kb full cDNA fragment; the pB4NU vector is 14.117 Kb (Mukherjee and Khurana, 2018) and uses restriction digestion/ligation as a cloning method, while the pBRACT214 is 9.165 kb (Rooke *et al*., 2000) and uses Gateway technology. The selected regions for silencing the gene are different, Borah and Khurana (2018) generated their *OsFBK1^KD^* lines using a 0.298Kb segment from the 3’ UTR region, while we use a non‐conserved region of 0.349 Kb in the 5’ end of the coding region. We have grown our plants in greenhouses or controlled chambers in the UK. If the expression and function of *OsFBK1* is affected by stress, it is possible that the morphology of anthers and other plant parts may be affected. Analysing the effect of the vectors, and the environmental conditions in the expression of *OsFBK1* may help explain the differences observed.

Our results support the hypothesis that *EP3* and *OsFBK1* effect floral organ development in rice and may be functional orthologues of the *HWS* gene in Arabidopsis (Gonzalez‐Carranza *et al*., 2007, 2017). Results also suggest that *EP3* and *OsFBK1* act antagonistically to influence the regulation of floral organ morphology, plant growth, architecture, flower development, pollen viability and yield in rice by targeting for degradation proteins that remain elusive.

### *EP3* and *OsFBK1* modulate transcript levels of *OsNAM*, *OsNAC1* and OsPri‐MIR164

We demonstrated that in Arabidopsis, *HWS* regulates size and floral organ number by modulating *CUC1* and *CUC2* transcript levels via *MIR164* (Gonzalez‐Carranza *et al*., 2017). The rice putative orthologue of the Arabidopsis *CUC1* and *CUC2* genes is *OsNAM* (Hibara and Nagato, 2005). In rice, there are six MIR164 (a–f) (Sunkar *et al*., 2008). Here we designed primers to detect and quantify all the Pri‐MIR164 from the six genes (Table S1).

**Figure 5:**
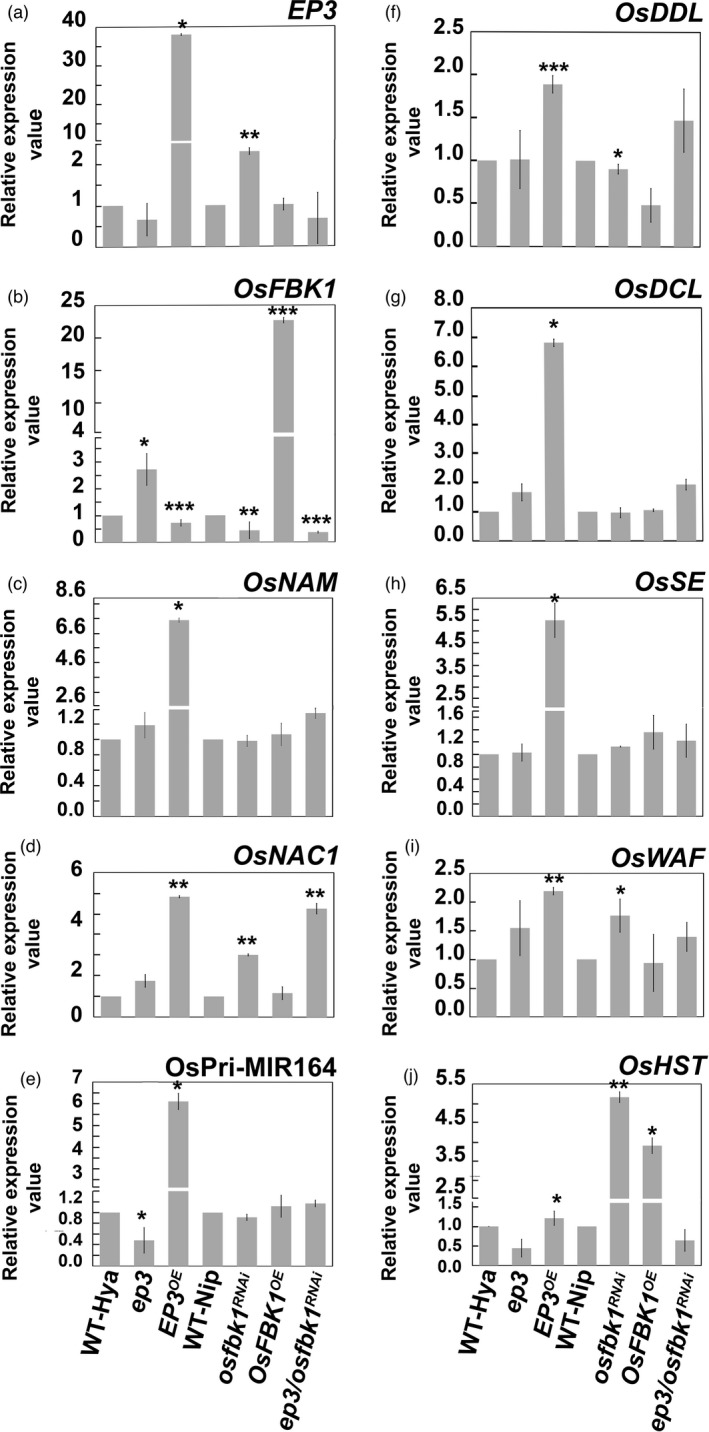
Transcript levels of *EP3*, *OsFBK1*, *OsNAM1*, *OsNAC1*, OsPri‐MIR164, *OsDDL*, *OsDCL*, *OsSE*, *OsWAF and OsHST* genes are affected in panicles (10–15 cm old from booting stage) from single, double and over expressor *EP3* and *OsFBK1* lines. RT‐qPCR measurements of (A), *EP3*; (B), *OsFBK1*; (C), *OsNAM*; (D), *OsNAC1*; (E), OsPri‐MIR164 (F) *OsDDL*; (G), *OsDCL;* (H), *OsSE*; (I), *OsWAF*, and (J) *OsHST* RNA levels in : WT‐Hya, *ep3*, *EP3^OE^*, WT‐Nip, *osfbk1^RNAi^*, *OsFBK1^OE^* and ep3/*osfbk1^RNAi^*. The *ep3*, *EP3^OE^* and ep3/*osfbk1^RNAi^* lines are in Hya background, while *osfbk1^RNAi^*, *OsFBK1^OE^* are in Nip background. Two sets of statistical analyses were done using WT Hya and WT Nip as controls for *EP3* and *OsFBK1* respectively. Stars indicate a significant difference in the mean at *P ≤ 0.05, **P ≤ 0.01 and ***P ≤ 0.001. Relative expression values represent the mean ± SD of three biological replicates from each sample (*n* = 21).

To determine if *EP3* and *OsFBK1* act in the same pathway as *HWS* to regulate size and floral organ number in rice, and to determine if the stress related gene *OsNAC1* (*ORIZYA SATIVA NO APICAL MERISTEM 1*) known to be regulated by *OsMIR164* (Chang *et al*., 2021; Fang *et al*., 2014) is affected by *EP3* and *OsFBK1*, we quantified transcript levels of *OsNAM*, *OsNAC1* and of OsPri‐MIR164 using qRT‐PCR. Significant increases of 6‐ to 6.5‐fold in the transcript levels for *OsNAM*, *OsNAC2* and OsPri‐MIR164 were observed in the *EP3^OE^* line (Figure 5C–E). This is expected and an over‐expression of OsPri‐MIR164 suggests little or no presence of mature *OsMIR164* regulatory transcripts. Therefore, up‐regulation of *OsNAM* and *OsNAC1* transcripts is observed. A significant decrease in transcript levels of OsPri‐MIR164 was observed in *ep3* (Figure 5E), however no significant increase of transcripts for *OsNAM* and *OsNAC1* was observed (Figure 5C,D), possibly because *ep3* is not completely silenced (Figure 5A), and enough mature *OsMIR164* is present to regulate the transcript levels of *OsNAM* and *OsNAC1*. Significant differences were observed in transcript levels of *OsNAC1* in *osfbk1* ^RNAi^ and in *ep3/osfbk1* ^RNAi^. However, the accumulation of *OsNAC1* transcript levels in *ep3/osfbk1* ^RNAi^ is greater than the sum of each independent *ep3* and *osfbk1* ^RNAi^ line, and instead a slight reduction in expression compared to *ep3* is observed, suggesting that *EP3* and *OsFBK1* regulate *OsNAC1* in an antagonistic manner (Figure 5D). No significant differences in *OsNAM* or OsPri‐MIR164 transcripts were observed in *osfbk1* ^RNAi^, *ep3/osfbk1* ^RNAi^ and *OsFBK1* ^OE^ lines. Transcript levels of *OsNAC1* were not affected in *OsFBK1* ^OE^ lines (Figure 5D). These results suggest that *EP3*, but not *OsFBK1*, positively affects the transcript levels of OsPri‐MIR164 and *OsNAM*. Transcript levels of *OsNAC1* moreover are regulated both by *EP3* and *OsFBK1* and an accumulative effect, which appears to be antagonistic, can be observed in the *ep3/osfbk1* ^RNAi^ line (Figure 5D).

Here we demonstrate that *EP3* positively influences transcript levels of OsPri‐MIR164 and *OsNAM*, while *OsNAC1* transcript levels are affected by both *EP3* and *OsFBK1*. These results suggest the presence of an alternative pathway in rice that influences *OsNAC1* transcripts independent of *MIR164*, where *OsFBK1* acts. Alternatively, *OsNAC1* maybe a target for OsFBK1. A protein–protein interaction experiment is needed to prove this hypothesis. *OsNAM* has been reported to be involved in regulating secondary branching of panicles and the number of spikelets under drought conditions (Kumar *et al*., 2014). *OsNAM* is also involved in rice leaf and panicle development (Chang *et al*., 2021). Our qRT‐PCR results help explain the phenotypes observed in our *EP3* transgenic lines. *OsNAC1*, is a rice stress‐sensitive gene (Fang *et al*., 2008; Khong *et al*., 2008). It is possible that *OsFBK1* and *EP3* may indirectly affect the regulatory mechanisms used by rice to regulate stress responses.

### *EP3* and *OsFBK1* affect transcript levels of genes involved in miRNA biogenesis

Our studies in Arabidopsis show that *HWS* alters transcript levels of genes from the microRNA pathway (Zhang *et al*., 2017). We hypothesized that *EP3* and *OsFBK1* affect transcript levels genes involved in this pathway in rice. To test this hypothesis, we used qRT‐PCR to analyse transcript levels of putative orthologues in rice known to be involved in biogenesis of microRNAs (Figure 5F–J). These include *OsDCL* (Liu *et al*., 2005) and *OsWAF* (Abe *et al*., 2010). We also identified and generated primers for qRT‐PCR of the putative rice orthologues of other genes likely to be involved in biogenesis and transport of miRNAs. We based our identification on sequence similarities to the Arabidopsis genes, previously reported to be involved in microRNA biogenesis. We named them following the Arabidopsis nomenclature. These include *OsDDL* (*Os05g0546600*) (International Rice Genome Sequencing Project, 2005), *OsSE (Os06g0698859)* (Kawahara *et al*., 2013; Sakai *et al*., 2013) and *OsHST* (*Os01g0363900*; Rice Full‐Length cDNA Consortium *et al*., 2003); named by Zhu *et al*. (2019) as *CROWN ROOT DEFECT 1* (CRD1). Significant increases in transcript levels of *OsDDL*, *OsDCL*, *OsSE*, *OsWAF* and *CRD1/OsHST* were observed in the *EP3^OE^* line (Figure 5F–J). Significant increases in transcript levels of *OsWAF* and *CRD1/OsHST* were observed in the *osfbk1* ^RNAi^ line (Figure 5I,J). Significant decreases or increases in transcript levels of *OsDDL* (Figure 5F) and *CRD1/OsHST* (Figure 5J) respectively were observed in *OsFBK1^OE^*. These results suggest that *EP3* and *OsFBK1* affect transcript accumulation of microRNA biogenesis genes, their transport and/ or their function in rice, likely by targeting a protein involved in the miRNA pathway.

Here we have identified the rice miRNA pathway putative orthologue genes *OsDDL*, *OsSE* and *OsHST*. Detailed analyses of these genes are necessary to confirm if indeed they perform similar functions as their orthologues from Arabidopsis in the rice miRNA pathway. Zhu *et al*., 2019 have demonstrated that in *CRD1/OsHST* 65% of miRNAs levels are down‐regulated, suggesting that *CRD1/OsHST* is necessary for proper miRNA accumulation. We have shown that transcript levels of *OsDDL*, *OsSE* and *OsHST* genes are altered in gain‐ and loss‐of‐function lines of *EP3* and *OsFBK1* as well as those of *OsDCL* (Liu *et al*., 2005; Song *et al*., 2012, 2013) and *OsWAF1* (Abe *et al*., 2010; Yu *et al*., 2005) genes. The data support the hypothesis that *EP3* and *OsFBK1* are involved in the miRNA pathway in rice. More analyses are necessary to determine their mode of action in such pathway. Many of the phenotypes described in this study may be regulated by microRNAs. *OsWAF1*, together with *SHOOT ORGANISATION1* (*DICER‐LIKE4* homologue), *SHOOTLESS2* (*RDR6* homologue) and *SHOOTLESS4* (*ARGONAUTE7* homologue) have been suggested to play a role in lemma and palea development (Abe *et al*., 2010; Toriba *et al*., 2010; Yoshida and Nagato, 2011). It will be interesting to investigate if these genes are affected in our *EP3 and OsFBK1* lines in future studies.

A question that arises from our investigations and the observations reported in the literature for *OsFBK1* is whether EP3 and OsFBK1 are targeting the same or different proteins for degradation. Landry *et al*. (2012) demonstrated that regulation of G1 cyclin levels in yeast is controlled by the two SCF ubiquitin ligases Cdc4 and Grr1 in a redundant manner related to the localization of the target in the nucleus or in the cytoplasm. They suggest the possibility of Cdc4 and Grr1 sharing additional redundant targets in their role to control cell cycle progression. Borah *et al*. (2021) have demonstrated that OsFBK1 targets for degradation OsCCR14 and the E3 ligase OsATL53. Other possible targets of OsFBK1 are yet to be elucidated. We have demonstrated that *HWS* affects the microRNA pathway (Zhang *et al*., 2017) and we report that *EP3* and *OsFBK1* may also be involved in the microRNA pathway in rice. Many important regulatory processes in cells are dependent on this pathway and tight regulation with a degree of flexibility is necessary for the survival of plants. More studies are necessary to prove if the functional redundancy of *EP3* and *OsFBK1* is present in rice and to define the targets of these genes.

## Materials and methods

Detailed methods for plant materials, and techniques are available in (Appendix S1). Arabidopsis and rice plants were grown in growth rooms, or glasshouses (rice) using compost, soil or hydroponics.

Primers used in this study are included in Table S1.

For complementing *hws‐1* with the coding region of *OsFBK1*, genomic DNA from Nip‐WT rice 10‐day‐old seedlings was extracted. The predicted coding region was amplified using primer set Rice1For/Rice1rev and cloned in the construct *Pro_HWS_:GUS* (Gonzalez‐Carranza *et al*., 2007).

Promoter reporter, RNAi knock out, and over‐expressing lines of *EP3* and *OsFBK1* were generated using Gateway® cloning technology (Hartley, 2003; Nakagawa *et al*., 2007). To generate *EP3_pro_:GFP/OsFBK1_pro_:RFP* plants, a simultaneous transformation of calli was performed as described by Nishiumra *et al*. (2006) and Zhou *et al*. (2003). *EP3_pro_:GFP*, *OsFBK1_pro_:RFP*, *OsFBK1^RNAi^* and *OsFBK1^OE^ EP3^OE^* lines were generated using WT‐Nip and WT‐Hya calli respectively. *ep3/osfbk1* ^RNAi^ was generated using *ep3* calli (WT‐Hya).

For *EP3* (WT‐Hya) and *OsFBK1* (WT‐Nip) expression analyses, total RNAs from 50‐day‐old roots, stems, leaves, panicles (10–20 cm) and young grains (milking stage) were extracted. For expression analyses of *OsNAM*, *OsNAC1*, OsPri‐MIR164, *OsDDL*, *OsDCL*, *OsSE*, *OsWAF* and *OsHST* total RNA was extracted from 10‐ to 15–cm‐long panicles of WT, *ep3*, *EP3^OE^* and *ep3/osfbk1* ^RNAi^ (Hya) and of WT, *osfbk1* ^RNAi^ and *OsFBK1^OE^* (Nip). Putative orthologues of *OsDDL*, *OsSE* and *OsHST* were identified by comparing Arabidopsis sequence genes from TAIR (Berardini *et al*., 2015), using a BLAST search in NCBI (Geer *et al*., 2010) or the rice genome annotation project (Kawahara *et al*., 2013) databases. Data analyses were performed using LightCycler® 480 Software 1.5 & Excel 365.

*EP3_pro_:GFP* and *OsFBK1_pro_:RFP* root (7‐day‐old plants), stems and leaf tissue (45‐day‐old plants) samples were embedded in 8% (v/v) agarose LMP (Helena Biosciences) and sectioned using a Ci 7000 vivratome (Campden instruments). *GFP* was excited using a 488nm line of multi argon ion laser and visualized between 500 and 530 nm. A combination of 488nm line of multi argon ion laser and 543nm line of a helium‐neon laser were used to excite *mRFP1*and visualized between 590 and 650 nm.

Glasshouse plants from WT‐Hya, and homozygous lines for *EP3*, *EP3^OE^*, WT‐Nip, *osfbk1* ^RNAi^, *OsFBK1* ^OE^ and *ep3/osfbk1* ^RNAi^ growing at the same time and under the same conditions, were used to analyse plant height, tiller number, and flag leaf dimensions. The same lines growing in hydroponics (Murchie *et al*., 2005) were used to analyse root length.

GFP and RFP expressions in calli and plant material were detected using either inverted Leica TCS SP6 confocal or Fluorescence Leica (Leica MZ10F) microscopes. Seed and floral morphology studies were performed using a dissecting stereomicroscope (Zeiss Stemi SV6). All measurements from microscopy images were performed using Fiji ImageJ (Schindelin *et al*., 2012). qRT‐PCR statistical analyses were performed using GenStat (17.1.0.14713). For other measurements, One Way ANOVA and Tukey HSD were done using Microsoft excel 2016 and GenStat (17.1.0.14713).

## Conflict of interest

The authors have declared that no conflict of interest exist.

## Author contributions

Z.H.G.C. conceptualized the project, conceived and designed the experiments; administered the project and funds; and wrote the manuscript. R.S.B. and Z.H.G.C. performed the experiments, curated and analysed the data, and prepared the figures. R.S.B, E.H.M and Z.H.G.C. acquired funds for this project. R.S.B, E.H.M, K.A.P, and Z.H.G.C. proposed the methodology. R.S.B, E.H.M, J.A.R. and Z.H.G.C. supervised the research. All authors, edited, read and approved the final version of the manuscript.

## Supporting information


**Figure S1** Complementation of *hws‐1* sepal fusion by *OsFBK1*.

**Figure S2** Panicles and seeds from loss‐ and gain‐of‐function lines from *EP3* and *OsFBK1*.

**Table S1** Primers used in this study.

**Appendix S1** Supplementary methods.

## References

[pbi13710-bib-0001] Abe, M. , Yoshikawa, T. , Nosaka, M. , Sakakibara, H. , Sato, Y. , Nagato, Y. and Itoh, J. (2010) *WAVY LEAF1*, an ortholog of Arabidopsis *HEN1*, regulates shoot development by maintaining MicroRNA and trans‐acting small interfering RNA accumulation in rice. Plant Physiol. 154, 1335–1346. 20805329 10.1104/pp.110.160234 PMC2971610

[pbi13710-bib-0002] Acosta, I. and Przybyl, M. (2019) Jasmonate signalling during *Arabidopsis* stamen maturation. Plant Scell. Physiol. 60, 2648–2659. 10.1093/pcp/pcz201 PMC6896695 31651948

[pbi13710-bib-0003] Aida, M. , Ishida, T. , Fukaki, H. , Fujishawa, H. and Tasaka, M. (1997) Genes involved in organ separation in *Arabidopsis*: an analysis of the *cuc*‐shaped cotyledon mutant. Plant Cell, 9, 841–857. 9212461 10.1105/tpc.9.6.841 PMC156962

[pbi13710-bib-0004] Berardini, T.Z. , Reiser, L. , Li, D. , Mezheritsky, Y. , Muller, R. , Strait, E. and Huala, E. (2015) The *Arabidopsis* information resource: making and mining the "gold standard" annotated reference plant genome. Genesis, 53, 474–485. 10.1002/dvg.22877 26201819 PMC4545719

[pbi13710-bib-0005] Borah, P. and Khurana, J.P. (2018) The OsFBK1 E3 ligase subunit affects anther and root secondary cell wall thickenings by mediating turn‐over of a cinnamoyl‐CoA reductase. Plant Physiol. 176, 2148–2165. 29295941 10.1104/pp.17.01733 PMC5841686

[pbi13710-bib-0006] Borah, P. , Sharma, A. and Khurana, P. (2021) The rice SCFOsFBK1 E3 ligase mediates jasmonic acid induced degradation of a RING‐H2 protein and the cinnamoyl‐CoA reductase, OsCCR14. March 2021. PREPRINT (Version 1) available at Research Square 10.21203/rs.3.rs-300884/v1

[pbi13710-bib-0007] Borah, P. , Sharma, E. , Kaur, A. , Chande, G. , Mohapatra, T. , Kapoor, S. and Khurana, J.P. (2017) Analysis of drought‐responsive signalling network in two contrasting rice cultivars using transcriptome‐based approach. Sci. Rep. 7, 42131. 28181537 10.1038/srep42131 PMC5299611

[pbi13710-bib-0008] Chang, Z. , Xu, R. , Xun, Q. , Liu, J. , Zhong, T. , Ding, Y. and Ding, C. (2021) OsmiR164‐targeted *OsNAM*, a boundary gene, plays important roles in rice leaf and panicle development. Plant J. 106, 41–55. 33368800 10.1111/tpj.15143

[pbi13710-bib-0009] Collins, G.A. and Goldberg, A.L. (2017) The logic of the 26S proteosome. Cell, 169, 792–806. 28525752 10.1016/j.cell.2017.04.023 PMC5609836

[pbi13710-bib-0010] Du, Z. , Zhou, X. , Li, L. and Su, Z. (2009) PlantsUPS: a database of plants' ubiquitin proteasome system. BMC Genom. 10, 227. 10.1186/1471-2164-10-227 PMC2690602 19445698

[pbi13710-bib-0011] Fang, Y. , Xie, K. and Xiong, L. (2014) Conserved miR164‐targeted *NAC* genes negatively regulate drought resistance in rice. J. Exp. Bot. 65, 2119–2135. 24604734 10.1093/jxb/eru072 PMC3991743

[pbi13710-bib-0012] Fang, Y. , You, J. , Xie, K. , Xie, W. and Xiong, L. (2008) Systematic sequence analysis and identification of tissue‐specific or stress‐responsive genes of NAC transcription factor family in rice. Mol. Genet. Genomics, 280, 547–563. 18813954 10.1007/s00438-008-0386-6

[pbi13710-bib-0013] Geer, L.Y. , Marchler‐Bauer, A. , Geer, R.C. , Han, L. , He, J. , He, S. , Liu, C. *et al*. (2010) The NCBI BioSystems database. Nucleic Acids Res. 38, D492–D496. 19854944 10.1093/nar/gkp858 PMC2808896

[pbi13710-bib-0014] González‐Carranza, Z.H. , Rompa, U. , Peter, J.L. , Bhatt, A. , Wagstaff, C. , Stead, A.D. and Roberts, J.A. (2007) *HAWAIIAN SKIRT* an F‐box gene that regulates organ fusion and growth in *Arabidopsis* . Plant Physiol. 144, 1370–1382. 17496113 10.1104/pp.106.092288 PMC1914148

[pbi13710-bib-0015] González‐Carranza, Z.H. , Zhang, X. , Peters, J. , Bolts, V. , Szecsi, J. , Bendahmane, M. and Roberts, J.A. (2017) *HAWAIIAN SKIRT* controls size and floral organ number by modulating *CUC1* and *CUC2* expression. PLoS One, 12, e0185106. 28934292 10.1371/journal.pone.0185106 PMC5608315

[pbi13710-bib-0016] Hartley, J. (2003) Use of the gateway system for protein expression in multiple hosts. Curr. Protoc. Protein Sci. 30, Chapter 5: Unit 5.17. 10.1002/0471140864.ps0517s30 18429245

[pbi13710-bib-0017] Hibara, K. and Nagato, Y. (2005) Petunia *NO APICAL MERISTEM* ortholog, *OsNAM*, is expressed in the embryonic SAM and organ boundary in rice. Rice Genet. Newslett. 22, 83.

[pbi13710-bib-0018] International Rice Genome Sequencing Project (2005) The map‐based sequence of the rice genome. Nature, 436, 793–800. 16100779 10.1038/nature03895

[pbi13710-bib-0019] Jain, M. , Nijhawan, A. , Arora, R. , Agarwal, P. , Ray, S. , Sharma, P. , Kapoor, S. *et al*. (2007) F‐box proteins in rice. Genome‐wide analysis, classification, temporal and spatial gene expression during panicle and seed development, and regulation by light and abiotic stress. Plant Physiol. 143, 1467–1483. 17293439 10.1104/pp.106.091900 PMC1851844

[pbi13710-bib-0020] Kawahara, Y. , de la Bastide, M. , Hamilton, J.P. , Kanamori, H. , McCombie, W.R. , Ouyang, S. , Schwartz, D.C. *et al*. (2013) Improvement of the *Oryza sativa* Nipponbare reference genome using next generation sequence and optical map data. Rice, 6, 4. 24280374 10.1186/1939-8433-6-4 PMC5395016

[pbi13710-bib-0021] Khong, G.N. , Richaud, F. , Coudert, Y. , Pati, P.K. , Santi, C. , Périn, C. , Breitler, J.C. *et al*. (2008) Modulating rice stress tolerance by transcription factors. Biotechnol. Genet. Eng. Rev. 25, 381–404. 21412363 10.5661/bger-25-381

[pbi13710-bib-0022] Kumar, A. , Dixit, S. , Ram, T. , Yadaw, R.B. , Mishra, K.K. and Mandal, N.P. (2014) Breeding high‐yielding drought‐tolerant rice: genetic variations and conventional and molecular approaches. J Exp. Bot. 65, 6265–6278. 25205576 10.1093/jxb/eru363 PMC4223988

[pbi13710-bib-0023] Landry, B.D. , Doyle, J.P. , Toczyski, D.P. and Benanti, J.A. (2012) F‐Box protein specificity for G1 cyclins is dictated by subcellular localization. PLoS Genet. 8, e1002851. 22844257 10.1371/journal.pgen.1002851 PMC3405998

[pbi13710-bib-0024] Lang, P.L.M. , Christie, M.D. , Dogan, E.S. , Schwab, R. , Hagmann, J. , Van De Weyer, A.‐L. , Scacchi, E. and *et al*. (2018) A role for the F‐box protein HAWAIIAN SKIRT in plant microRNA function. Plant Physiol. 176, 730–741. 29114080 10.1104/pp.17.01313 PMC5761791

[pbi13710-bib-0025] Laufs, P. , Peaucelle, A. , Morin, H. and Traas, J. (2004) MicroRNA regulation of the *CUC* genes is required for boundary size control in *Arabidopsis* meristems. Development, 131, 4311–4322. 15294871 10.1242/dev.01320

[pbi13710-bib-0026] Li, M. , Tang, D. , Wang, K. , Wu, LuL , Yu, H. , Gu, M. , Yan, C. and *et al*. (2011) Mutations in the F‐box gene *LARGER PANICLE* improve the panicle architecture and enhance the grain yield in rice. Plant Biotech. J. 9, 1002–1013. 10.1111/j.1467-7652.2011.00610.x 21447055

[pbi13710-bib-0027] Liu, B. , Li, P. , Li, X. , Liu, C. , Cao, S. , Chu, C. and Cao, X. (2005) Loss of function of *OsDCL1* affects microRNA accumulation and causes developmental defects in rice. Plant Physiol. 139, 296–305. 16126864 10.1104/pp.105.063420 PMC1203379

[pbi13710-bib-0028] Liu, Q. and Chen, Y.‐Q. (2009) Insights into the mechanism of plant development: interactions of miRNAs pathway with phytohormone response. Bioch. Biophys. Res. Commun. 384, 1–5. 10.1016/j.bbrc.2009.04.028 19366618

[pbi13710-bib-0029] Mallory, A.C. , Dugas, D.V. , Bartel, D.P. and Bartel, B. (2004) MicroRNA regulation of NAC‐domain targets is required for proper formation and separation of adjacent embryonic, vegetative, and floral organs. Curr. Biol. 14, 1035–1046. 15202996 10.1016/j.cub.2004.06.022

[pbi13710-bib-0030] Mallory, A.C. and Vaucheret, H. (2006) Functions of microRNAs and related small RNAs in plants. Nat. Genet. 38, S31ÐS36. 16736022 10.1038/ng1791

[pbi13710-bib-0031] Mukherjee, S. and Khurana, J.P. (2018) Developmental defects in Pusa Basmati 1 transgenic rice plants harboring antisense OsiEZ1 gene. Int. J. Curr. Res. Biosci. Plantbiol. 5, 53–69.

[pbi13710-bib-0032] Murchie, E.H. , Hubbart, S. , Peng, S. and Horton, P. (2005) Acclimation of photosynthesis to high irradiance in rice: gene expression and interactions with leaf development. J. Exp. Bot. 56, 449–460. 15647315 10.1093/jxb/eri100

[pbi13710-bib-0033] Nakagawa, T. , Suzuki, T. , Murata, S. , Nakamura, S. , Hino, T. , Maeo, K. , Tabata, R. *et al*. (2007) Improved gateway binary vectors: high‐performance vectors for creation of fusion constructs in transgenic analysis of plants. Biosci. Biotechnol. Biochem. 71, 2095–2100. 17690442 10.1271/bbb.70216

[pbi13710-bib-0034] Nishimura, A. , Aichi, I. and Matsuoka, M. (2006) A protocol for *Agrobacterium*‐mediated transformation in rice. Nat. Protoc. 1, 2796–27802. 17406537 10.1038/nprot.2006.469

[pbi13710-bib-0035] Petroski, M.D. and Deshaies, R.J. (2005) Function and regulation of cullin‐RING ubiquitin ligases. Nat. Rev. Mol. Cell Biol. 6, 9–20. 15688063 10.1038/nrm1547

[pbi13710-bib-0036] Piao, R. , Jiang, W. , Ham, T.H. , Choi, M.S. , Qiao, Y. , Chu, S.H. , Park, J.H. *et al*. (2009) Map‐based cloning of the *ERECT PANICLE 3* gene in rice. Theor. Appl. Genet. 119, 1497–1506. 19756471 10.1007/s00122-009-1151-x

[pbi13710-bib-0037] Reed, S.I. (2003) Ratchets and clocks: the cell cycle, ubiquitylation and protein turnover. Nat. Rev. Mol. Cell Biol. 4, 855–864. 14625536 10.1038/nrm1246

[pbi13710-bib-0038] Rice Full‐Length cDNA Consortium (2003) Collection, mapping, and annotation of over 28,000 cDNA clones from Japonica rice. Science, 301, 376–379. 12869764 10.1126/science.1081288

[pbi13710-bib-0039] Rooke, L. , Byrne, D. and Salgueiro, S. (2000) Marker gene expression driven by the maize ubiquitin promoter in transgenic wheat. Ann. Appl. Biol. 136, 167–172.

[pbi13710-bib-0040] Sakai, H. , Lee, S.S. , Tanaka, T. , Numa, H. , Kim, J. , Kawahara, Y. , Wakimoto, H. *et al*. (2013) Rice annotation project database (RAP‐DB): an integrative and interactive database for rice genomics. Plant Cell Physiol. 54, e6. 23299411 10.1093/pcp/pcs183 PMC3583025

[pbi13710-bib-0041] Schindelin, J. , Arganda‐Carreras, I. , Fris, E. , Kaynig, V. , Longair, M. , Pietzsch, T. , Preibisch, S. *et al*. (2012) Fiji: an open‐source platform for biological‐image analysis. Nat. Meth. 9, 676–682. 10.1038/nmeth.2019 PMC3855844 22743772

[pbi13710-bib-0042] Sharma, B. , Joshi, D. , Yadav, P.K. , Gupta, A.K. and Bhatt, T.K. (2016) Role of ubiquitin‐mediated degradation system in plant biology. Front. Plant Sci. 7, 806. 27375660 10.3389/fpls.2016.00806 PMC4897311

[pbi13710-bib-0043] Song, X. , Li, P. , Zhai, J. , Zhou, M. , Ma, L. , Liu, B. , Jeong, D.H. *et al*. (2012) Roles of DCL4 and DCL3b in rice phased small RNA biogenesis. Plant J. 69, 462–474. 21973320 10.1111/j.1365-313X.2011.04805.x

[pbi13710-bib-0044] Song, X. , Liu, X. , Cao, X. and Wang, X.J. (2013) Noncoding regulatory RNAs. In: Genetics and Genomics of Rice ( Zhang, Q. and Wing, R.A. , eds), pp. 145–160. New York: Springer. 145

[pbi13710-bib-0045] Sugiyama, S.‐H. , Yasui, Y. , Ohmori, S. , Tanaka, W. and Hirano, H.‐Y. (2019) Rice flower development revisited: regulation of carpel specification and flower meristem determinacy. Plant Cell Physiol. 60, 1284–1295. 30715478 10.1093/pcp/pcz020

[pbi13710-bib-0046] Sunkar, R. , Zhou, X. , Zheng, Y. , Zhang, W. and Zhu, J.K. (2008) Identification of novel and candidate miRNAs in rice by high throughput sequencing. BMC Plant Biol. 8, 25. 18312648 10.1186/1471-2229-8-25 PMC2292181

[pbi13710-bib-0047] Toriba, T. , Suzaki, T. , Yamaguchi, T. , Ohmori, Y. , Tsukaya, H. and Hirano, H.Y. (2010) Distinct regulation of Adaxial‐abaxial polarity in anther patterning in rice. Plant Cell, 22, 1452–1462. 20511295 10.1105/tpc.110.075291 PMC2899876

[pbi13710-bib-0048] Yang, Y. , Zhu, K. , Xia, H. , Chen, L. and Chen, K. (2014) Comparative proteomic analysis of Indica and Japonica rice varieties. Genet. Mol. Biol. 37, 652–661. 25505840 10.1590/S1415-47572014005000015 PMC4261965

[pbi13710-bib-0049] Yoshida, H. and Nagato, Y. (2011) Flower development in rice. J. Exp. Bot. 62, 4719–4730. 21914655 10.1093/jxb/err272

[pbi13710-bib-0050] Yu, B. , Yang, Z. , Li, J. , Minakhina, S. , Yang, M. , Padgett, R.W. , Steward, R. and *et al*. (2005) Methylation as a crucial step in plant microRNA biogenesis. Science, 307, 932. 15705854 10.1126/science.1107130 PMC5137370

[pbi13710-bib-0051] Yu, H. and Matouschek, A. (2017) Recognition of client proteins by the proteasome. Annu. Rev. Biophys. 46, 149–1473. 28301771 10.1146/annurev-biophys-070816-033719

[pbi13710-bib-0052] Yu, H. , Murchie, E.H. , Gonzalez‐Carranza, Z.H. , Pyke, K.A. and Roberts, J.A. (2015) Decreased photosynthesis in the erect panicle 3 (ep3) mutant of rice is associated with reduced stomatal conductance and attenuated guard cell development. J. Exp. Bot. 66, 1543–1552. 25582452 10.1093/jxb/eru525 PMC4339609

[pbi13710-bib-0053] Zhang, X. , Jayaweera, D. , Peters, J.L. , Szecsi, J. , Bendahmane, M. , Roberts, J.A. and González‐Carranza, Z.H. (2017) The *Arabidopsis thaliana* F‐box gene HAWAIIAN SKIRT is a new player in the microRNA pathway. PLoS One, 12, e0189788. 29244865 10.1371/journal.pone.0189788 PMC5731758

[pbi13710-bib-0054] Zhang, X. , Zhanga, X. , Gonzalez‐Carranza, Z.H. , Zhanga, S. , Miaoa, Y. , Liuc, C.‐J. and Roberts, J.A. (2019) F‐box proteins in plants. Annu. Plant Rev. 2, 1–21. 10.1002/9781119312994.apr0701

[pbi13710-bib-0055] Zhou, H.‐Y. , Songbiao, C.H. , Xugang, L. , Guifang, X. , Xiaoli, W. and Zhen, Z. (2003) Generating marker free transgenic tobacco plant by *Agrobacterium* mediated transformation with double T‐DNA Binary vector. Acta Botanica Sinica, 45, 1103–1108.

[pbi13710-bib-0056] Zhu, J. , Li, Y. , Lin, J. , Wu, Y. , Guo, H. , Shao, Y. , Wang, F. *et al*. (2019) CRD1, an Xpo1 domain protein, regulates miRNA accumulation and crown root development in rice. Plant J. 100, 328–342. 31257621 10.1111/tpj.14445

