# Supplementary figures and images for: The rice EP3 and OsFBK1 E3 ligases alter plant architecture and flower development, and affect transcript accumulation of microRNA pathway genes and their targets
Source: Plant Biotechnol J. 2021 Oct 1;20(2):297–309. doi: 10.1111/pbi.13710 (PMC8753360; doi:10.1111/pbi.13710)

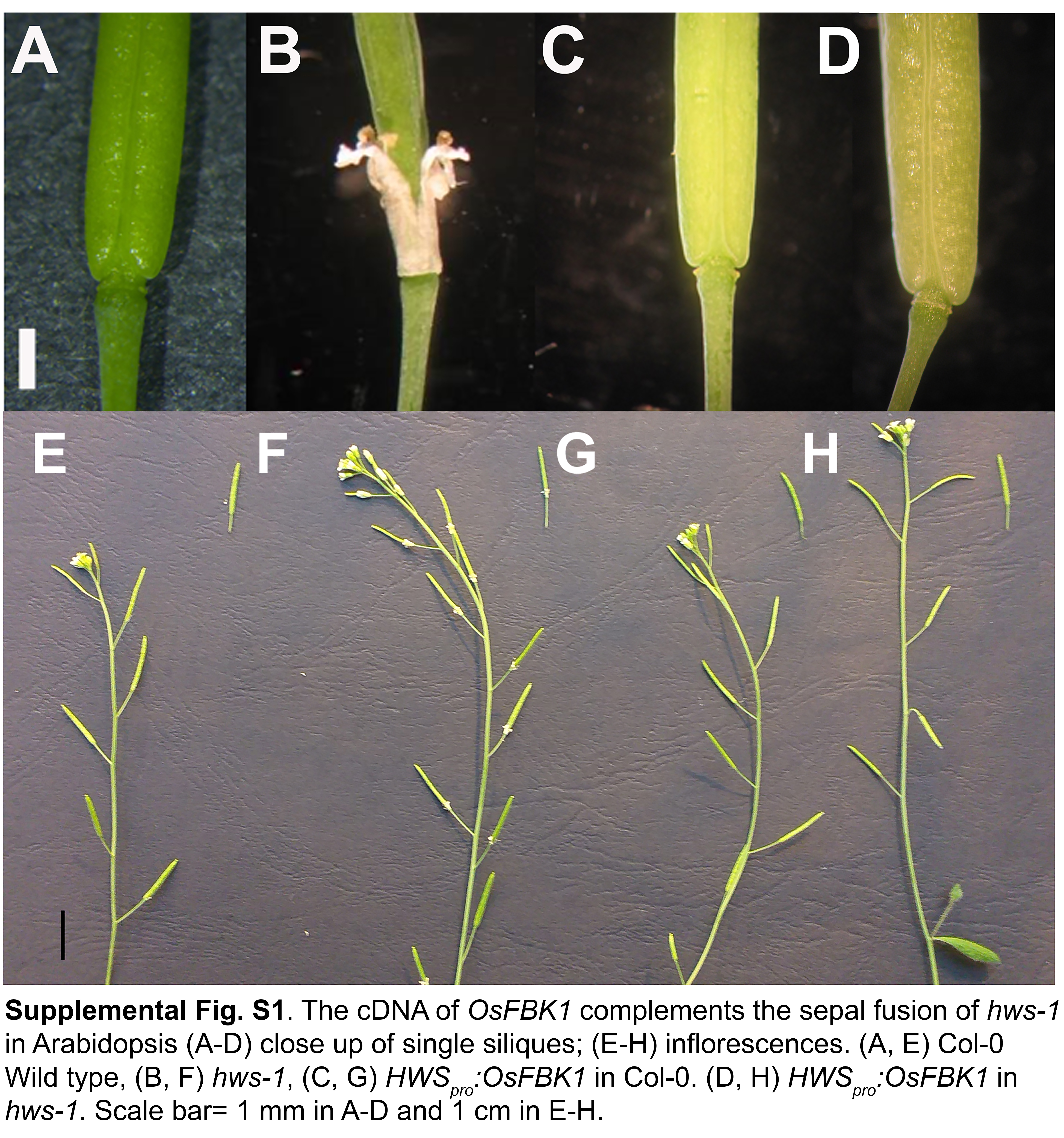

Supplement: Supplementary file 1 — Figure S1 Complementation of hws‐1 sepal fusion by OsFBK1. [file PBI-20-297-s001.jpg]

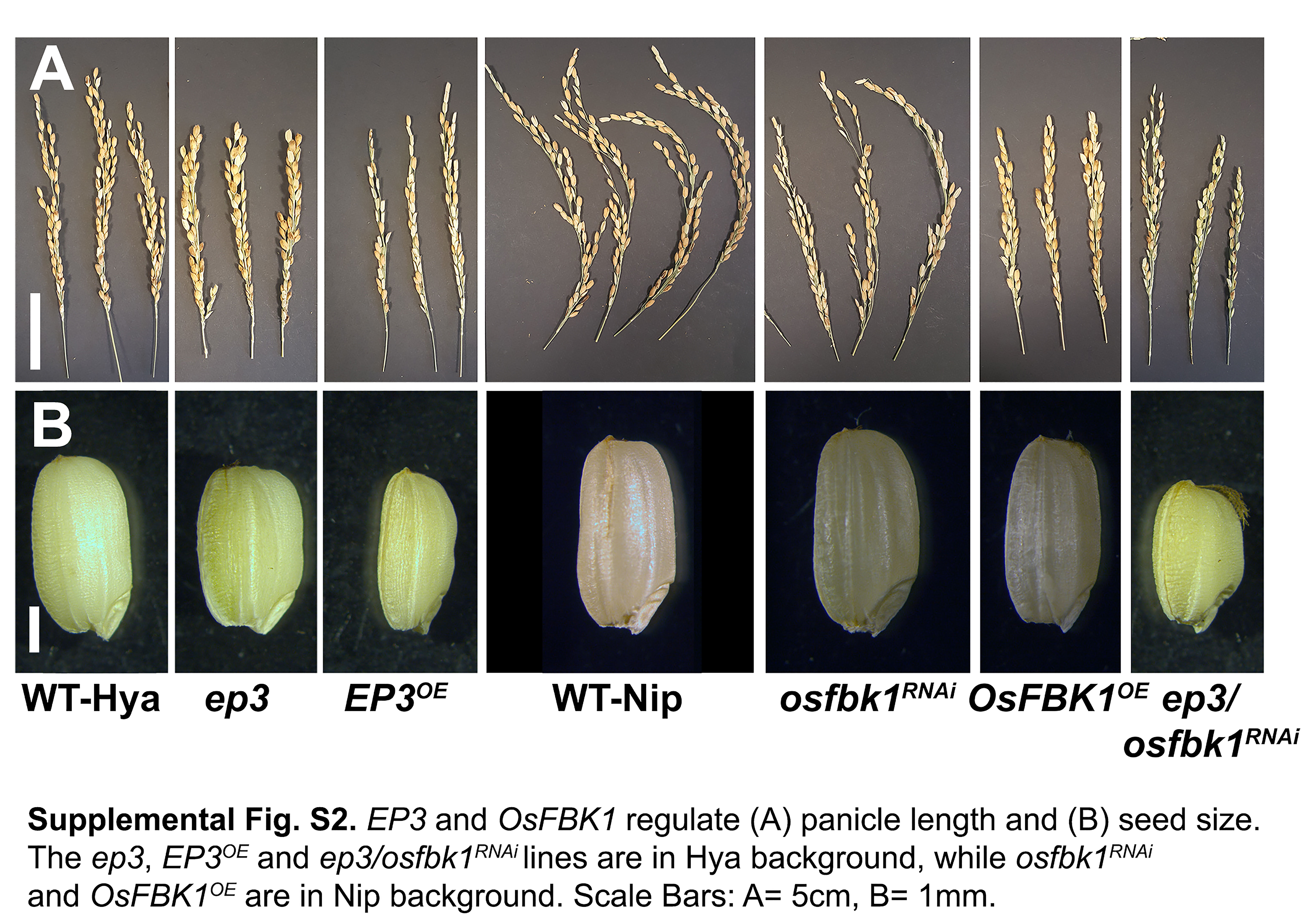

Supplement: Supplementary file 2 — Figure S2 Panicles and seeds from loss‐ and gain‐of‐function lines from EP3 and OsFBK1. [file PBI-20-297-s003.jpg]
