# Supplementary material for: The rice EP3 and OsFBK1 E3 ligases alter plant architecture and flower development, and affect transcript accumulation of microRNA pathway genes and their targets
Source: Plant Biotechnol J. 2021 Oct 1;20(2):297–309. doi: 10.1111/pbi.13710 (PMC8753360; doi:10.1111/pbi.13710)
Supplement: Supplementary file 3 — Table S1 Primers used in this study. [file PBI-20-297-s002.docx]

**Supplemental Table 1. Primers used in this study.**

| Experiment | Primer name | Sequence (5’ to 3’) |
| --- | --- | --- |
| *OsFBK1* cloning to transform *hws-1*, generation of *OsFBK1^OE^* and transcript analyses | OsFBK1cDNAFor | ATGCTAGCACACTTGATGACTG |
|  | OsFBK1cDNArev | CAATTAAGTGGTAATATCAAGCCGAG |
| *EP3* promoter amplification | Ep3proFor | CCATGATGTTGTCGCATTCGGCAC |
|  | Ep3proRev | CCAGCGAACAGCACAGTAACTAAC |
| *OsFBK1* promoter amplification | OsHWSProFor2 | CAGCGTTGTGGTACGAAACCGAG |
|  | OsHWSproRev | AGATGACCTGAATAAAAAAAAGTTATATTTATAG |
|  | OsHWS-Intron-ORF- | CTTGCTCGTGCTGTTCTAATG |
| *EP3* sequence confirmation of clones | OsEP3ProclFOR | TTCGGCACTTCTCCCTTGAC |
|  | EP3 Intron-ORF For | CTAGATTAGATGTTACCATGTGG |
|  | EP3-CMRev | GGAAGCAATGTCCAGCCTAGGC |
|  | Rice-EGFP-Rev | GTGAACAGCTCCTCGCCCTTGC |
| *OsFBK1* sequence confirmation of clones | OsHWSProclREV | CCAACGGACAATGTACCCGA |
|  | OsHWS-CM-Rev | AGTGGTAATATCAAGCCGAGGTTC |
| *OsFBK1* silencing construct by RNAi | OsHWS-RNAi-for | CACTTGATGACTGATGTCATGG |
|  | OsHWS-RNAi-  Rev | GCGGAGAATCGGGTCATATGC |
| PCR and sequencing confirmation, vector specific primers | pAHUbipromD for | GGCATATGCAGCAGCTATATGTG |
|  | nosterm_3' Rev | GATATCAGCTTGCATGCCGGTC |
|  | RFP Rev | GTACTGTTCCACGATGGTG |
| Gene expression analysis and cloning | Rice-2 For | ATGCTGGCAATGGGGTCAGAGG |
|  | Rice-2 Rev | CTAGGAAGCAATGTCCAGCCTAG |
| TOPO cloning Sequence confirmation | M13 For | TGTAAAACGACGGCCAG |
| qRT analysis | F2 EP3  R2 EP3-Reverse  OsHWS-orf-for  OsHWS-orf-rev  OsEP3-orf-for  OsEP3-orf-rev  Os-SE for  Os-SE rev  OsDCL-1for  OsDCL-1rev  OsWAF1(HEN-1)for  OsWAF1(HEN-1)rev  OsHASTY1 for  OsHASTY1 rev  OsCUC1/2 for  (OsNAM)  OsCUC1/2 rev  (OsNAM)  OsNAC1 for  OsNAC1 rev  OsMir164b for  OsMir164b rev  eEF-1α For  eEF-1α Rev | CATGGGTGTCTCCCTTCACT  GGTGAATGGCATCGTTTGAA  CACACTTGATGACTGATGTC  GATATCATCAGGAACAACAGTG  CCATCTTCCTTCATTGGTGC  CTTATGACACTTGCAACAGGC  CCTTCGCCGCCGTTCAAG  CTGCCATGGTATCGGTCATC  CTCAAGGAGAACAAGAAGATG  CACTGCATGATGGCACTC  AATCATGCGTGAGTCAGCTG  CATTCTATCTTCTAATGGTTCAG  GGA ACT CAG TGT TGC AGA AC  CGA AAC AAT AGA TGG GAG TAA AG  GTCATGCACGAGTACCGC  GAGAAGCACGAGGTGTCG  ACAATATGGGTAGGGCGATCAA  CACATTTGTGTGAAGCCACTC  GGAGAAGCAGGGCACGTGCA  GTG CTC GGT GG AAA AAA AAA AAA AAA AAA AAA  TTTCACTCTTGGTGTGAAGCAGAT  GCCTCTTGGGCTCGTTGATC |
