# Supplementary material for: The rice EP3 and OsFBK1 E3 ligases alter plant architecture and flower development, and affect transcript accumulation of microRNA pathway genes and their targets
Source: Plant Biotechnol J. 2021 Oct 1;20(2):297–309. doi: 10.1111/pbi.13710 (PMC8753360; doi:10.1111/pbi.13710)
Supplement: Supplementary file 4 — Appendix S1 Supplementary methods. [file PBI-20-297-s004.docx]

**Appendix S1 Supplementary methods**

**Plant materials and growth conditions**

Arabidopsis Col-0 WT where sourced from NASC. The *hws-1* seeds were identified as described by Gonzalez-Carranza et al., 2007. Plants were grown in a room with a temperature of 22±2 ̊ C, and photoperiod of 22h light/2h darkness supplemented with fluorescent lights at a light intensity of 200 μmol m-2s-1 (Polylox XK 58W G-E 93331).

During summertime, rice plants were maintained in a glasshouse with a temperature of 28˚C ± 3 ˚C; a photoperiod of 12/12h dark/ light and humidity between 60%-70%. Seeds were germinated and grown in a mix of 50% Levington M3 compost, and 50% John Innes 3 compost soil. Plants were transplanted 10 days after germination and kept under continuous water submergence. Plants were supplemented twice during the growing season with Osmocote® fertilizer (14-14-14NPK). Rice plants grown in hydroponic solution (Murchie et al., 2005), were maintained in a growth room with temperature of 28˚C / 26˚C day/night; a photoperiod of 12/12h dark/ light with light intensity of 600 µmol m^–2^ s^–1^ at canopy height, and 60% humidity.

**Construction of plasmids and plant transformation**

All DNA and total RNA used in this study were isolated using GenElute Plant Genomic DNA Miniprep (SIGMA-ALDRICH) and SV Total RNA Isolation System (Promega) kits respectively. PCR products were purified using Gene Jet PCR purification kit (Thermo Scientific). For all Gateway reactions, clones were prepared using the PCR/8/GW/TOPO TA Cloning Kit and *E. coli* Top10 competent cells (Thermo Fisher Scientific); manufacturer’s instructions were followed accordingly when using these kits.

For complementing the *hws-1* Arabidopsis mutant with the coding region of *OsFBK1,* genomic DNA from rice WT-Nip was extracted and the predicted coding region amplified using the primers Rice1For and Rice1rev (Table S1). PCR reactions were performed using Platinum1 pfx DNA polymerase (Invitrogen). The amplified band was gel purified using Genelute™ Gel extraction kit (Sigma) and confirmed by sequencing it. The *OsFBK1* coding region was cloned by substituting the GUS gene in the construct *Pro_HWS_:GUS* previously described (Gonzalez-Carranza et al., 2007).

To generate promoter reporter, RNAi knock out, and overexpressing plants of *EP3* and *OsFBK1,* Gateway® cloning technology (Hartley, 2003) was used to generate the constructs. Plants were transformed using Agrobacterium (GV3101 strain). For promoter reporter lines, the constructs *EP3_pro_:GFP* and *OsFBK1_pro_:RFP* were prepared using genomic DNA from rice WT-Nip. Bands of 2.686 Kb (including promoter, intron, 5’UTR and a segment of the coding region) and 2.739 Kb (including promoter, intron and 5’UTR) respectively were amplified using EP3proFor/EP3proRev and OsHWSProFor2 /OsHWSproRev primer sets respectively (Table S1). Clones for rice callus transformation were prepared in pGHGWG (*EP3_pro_:GFP)* (Zhong et al., 2008) and pGWB453 (*OsFBK1_pro_:RFP*) (Nakagawa et al., 2007) vectors after confirmation of correct orientation of segments cloned.

After careful *EP3* and *OsFBK1* sequence examination, a low identity region of 0.348 Kb was identified and amplified using the OsHWS-RNAi-for/OsHWS-RNAi-rev primer set (Table S1). pBract207 destination vector was used for this clone. (https://www.jic.ac.uk/technologies/ genomic-services/bract/constructs/).

The constructs *EP3^OE^* and *OsFBK1^OE^* were generated using the pBRACT214 vector, which constitutively expresses the maize Ubiquitin 1 gene (Rooke et al., 2000). Segments of 1.227Kb and 1.236Kb coding regions of *EP3* and *OsFBK1* respectively were amplified from total RNA using Rice-2For/ Rice-2Rev and Rice-1For/ Rice-1Rev set of primers respectively (Table S1).

Correct orientation of cloned products was confirmed by PCR and sequencing using vector and insert specific primers for each construct (Table S1).

Rice callus transformation was performed as described by (Toki et al., 2006) using 3-4 week-old callus (Nishimura et al 2006). To generate *EP3_pro_:GFP/OsFBK1_pro_:RFP* plants, a simultaneous transformation of callus was performed following the protocol described by Zhou, 2003. *EP3_pro_:GFP,* *OsFBK1_pro_:RFP*, *EP3_pro_:GFP & OsFBK1_pro_:RFP, OsFBK1^RNAi^* and *OsFBK1^OE^*; and *EP3^OE^* lines were generated using callus from WT-Nip plants and WT-Hya. Respectively. *ep3/osfbk1*^RNAi^ was generated using callus from *ep3* mutant in WT-Hya. To select positive callus, calli were screened under a florescent dissecting microscope (Leica MZ10F).

**qRT-PCR analyses of gene expression**

Total RNAs from roots (50 day-old plants), stems (seven day-old plants), leaves (leaf number 6), panicles (15-20 cm) and grains (milk stage) from three homozygous plants of WT-Hya for *EP3* and WT-Nip for *OsFBK1* expression were extracted. For analyses of expression of *OsNAM, OsNAC1, OSMIR164, OsDDL, OsDCL, OsSE, OsWAF* and *OsHST* total RNA was extracted from 10-15 cm long panicles of WT, *ep3, EP3^OE^* and *ep3/osfbk1*^RNAi^ (Hya background) and of WT, *osfbk1*^RNAi^ and *OsFBK1^OE^* (Nip background). The putative orthologues *OsDDL, OsSE* and *OsHST* were identified by comparing the sequences from the Arabidopsis genes obtained from the TAIR (Berardini et al., 2015) and performed a BLAST search in NCBI (Geer et al., 2010) or the rice genome annotation project (Kawahara et al., 2013) databases. Sequences of predicted coding regions from genes with the closest homology were selected to generate primers.

Three biological replicates from each sample were used in qRT-PCR analyses (n=21). Reverse transcription of total RNA was performed using M-MuLV Reverse Transcriptase following manufacturer’s instructions (New England Biolab). qRT-PCR reactions were performed as described by Gonzalez-Carranza et al., 2017. Quantification of transcripts was done using the *eEF-1α* rice gene as endogenous reference. Data analyses were performed using LightCycler® 480 Software 1.5 and Excel 365. Primers for qRT-PCR are included in Table S1. Statistical analyses were performed using GenStat (17.1.0.14713) and graphs generated with Microsoft Excel 2016 and annotated in Adobe Photoshop 7.0.1.

**Confocal and Fluorescent Microscopy**

GFP and RFP expressions driven by the *EP3* and the *OsFBK1* promoters were detected using either inverted Leica TCS SP6 confocal or Fluorescence Leica (Olympus SZX9, LEICA DM500 or LEICA DFC420) microscopes. GFP was excited using a 488nm line of multi argon ion laser and visualised between 500 to 530 nm. A combination of 488nm line of multi argon ion laser and 543nm line of a helium-neon laser were used to excite mRFP1and visualised between 590 to 650 nm. Root, stems and leaf tissue samples from T0 plants from *EP3_pro_:GFP* and *OsFBK1_pro_:RFP* lines were embedded in 8% (v/v) agarose LMP (Helena Biosciences) and sectioned using a Ci 7000 vivratome (Campden instruments); leaf samples were taken from the middle part of a fully expanded flag leaf and mounted in 10 μl of water prior to imaging. Floral organs from *EP3_pro_:GFP* and *OsFBK1_pro_:RFP* were mounted using 2% (v/v) glycerol; and stigma and stamens mounted in 20 μl of sterile distilled water. Developing grains from *EP3_pro_:GFP* and *OsFBK1_pro_:RFP* were collected from mature spikelets (milk stage spikelet from 70 days-old plants). All the images were recorded using LEICA microscopes (TCS SP6 confocal microscope, LEICA MZ10F, LEICA DM500 or LEICA DFC420) and analysed using Fiji ImageJ software (Schindelin et al., 2012).

**Plant architecture, yield and floral analyses**

Plants from WT-Hya, *EP3*, *EP3^OE^*, WT-Nip, *osfbk1*^RNAi^, *OsFBK1*^OE^ and *ep3/osfbk1*^RNAi^ lines growing under glasshouse conditions were used to analyse plant height (n=51), tiller number (n=56), and flag leaf dimensions (n=56). The same lines growing in hydroponics were used to analyse root length (n=51). Plant height was recorded at 14, 21, 60 and 90 days. Tiller number was analysed from 60 day old plants. Flag leaf dimensions were taken from fully mature expanded flag leaves of 90 day old plants and root analyses were taken at 14 and 21 days of development. Five mature panicles from 5 plants from each line: WT-Hya, *EP3*, *EP3^OE^*, WT-Nip, *osfbk1*^RNAi^, *OsFBK1*^OE^ and *ep3/osfbk1*^RNAi^ were analysed. The length, total number of seed per panicle and total number of filled grains per panicle were recorded, n=35. Length, width and area of ten grains from each line were noted n=700­­­­­­; and the dry weight of 100 grains from each line was logged. To study the effects of *EP3* and the *OsFBK1*in floral morphology, three fully mature flowers from three different plants (n=63) from the WT-Hya, *EP3*, *EP3^OE^*, WT-Nip, *osfbk1*^RNAi^, *OsFBK1*^OE^ and *ep3/osfbk1*^RNAi^ lines were dissected; and their floral organs counted, measured and recorded. Pollen viability was determined studying three flowers from each line n=21. Seed and floral morphology studies were performed using a dissecting stereomicroscope (Zeiss Stemi SV6). Pictures were taken using an AxioCam ERc 5s camera. All measurements from microscopy images were performed using Fiji ImageJ (Schindelin et al., 2012). Statistical analyses were performed using One Way Anova, Tukey HSD and linear regression analyses. Plant architectures analyses were done using Microsoft excel 2016 and GenStat (17.1.0.14713) (VSN International, 2019). Pictures were annotated using Adobe Photoshop 7.0.1.

**Accession numbers**

Sequence data can be found in the Arabidopsis Genome Initiative, NCBI databases, the Rice Genome Database, and GenBank under the following accession numbers: *HWS*, *At3g61590*; *EP3, Os02g15950; OsFBK1, Os01g47050; OsNAM, Os06g0344900; OsNAC1,* *Os06g0675600; OsMIR164 (OsMIR164a* [*HM139196*], *OsMIR164b* [*Os05g0339300*], Os*MIR164c* [*HM139239*], *OsMIR164d* [*HM139281*], *OsMIR164e* [*HM139304*]. *OsMIR164f* [*LM379362*]*; OsDCL,* *Os03g0121800; OsWAF1,* *Os07g0164000; OsDDL, Os05g0546600; OsSE, Os06g0698859;* and *OsHST, Os01g0363900.*

**Supplementary references:**

Berardini TZ, Reiser L, Li D, Mezheritsky Y, Muller R, Strait E, Huala E. (2015). The Arabidopsis Information Resource: Making and mining the "gold standard" annotated reference plant genome. *Genesis* doi: 10.1002/dvg.22877.

Geer LY, Marchler-Bauer A, Geer RC, Han L, He J, He S, Liu C, Shi W, Bryant SH. (2010). The NCBI BioSystems database. *Nucleic Acids Res*. **38**: D492-6.

González-Carranza ZH, Rompa U, Peter JL, Bhatt A, Wagstaff C, Stead AD, Roberts JA. (2007) *HAWAIIAN SKIRT* an F-box gene that regulates organ fusion and growth in Arabidopsis. *Plant Physiol* **144**: 1370- 1382.

González-Carranza ZH, Zhang X, Peters J, Bolts V, Szecsi J, Bendahmane M, Roberts JA. (2017). *HAWAIIAN SKIRT* Controls Size and Floral Organ Number by Modulating *CUC1* and *CUC2* expression. *PLOSONE* **12**:e0185106.

Hartley J (2003). Use of the Gateway System for Protein Expression in Multiple Hosts. *Curr Protoc Protein Sci.*; Chapter 5:Unit 5.17. doi: 10.1002/0471140864.ps0517s30

Kawahara Y, de la Bastide M, Hamilton JP, Kanamori H, McCombie WR, Ouyang S, Schwartz DC, Tanaka T, Wu J, Zhou S,Childs KL, Davidson RM, Lin H, Quesada-Ocampo L, Vaillancourt B, Sakai H, Lee SS, Kim J, Numa H, Itoh T, Buell CR, Matsumoto T. (2013). Improvement of the *Oryza sativa* Nipponbare reference genome using next generation sequence and optical map data. Rice (N Y). 6 (1): 4.

Murchie EH, Hubbart S, Peng S, Horton P. (2005). Acclimation of photosynthesis to high irradiance in rice: gene expression and interactions with leaf development. *J Exp Bot* **56**: 449-460.

Nakagawa T, Suzuki T, Murata S, Nakamura S, Hino T, Maeo K, Tabata R, Kawai T, Tanaka K, Niwa Y, Watanabe Y, Nakamura K, Kimura T, Ishiguro S. (2007). Improved Gateway Binary Vectors: High-Performance Vectors for Creation of Fusion Constructs in Transgenic Analysis of Plants. *Biosci Biotechnol Biochem* **71**: 2095-2100.

Nishimura A, Aichi I, Matsuoka M. (2006). A protocol for *Agrobacterium*-mediated transformation in rice. *Nat Protoc* **1**:2796-27802.

Rooke L, Byrne D, Salgueiro S. (2000). Marker gene expression driven by the maize ubiquitin promoter in transgenic wheat. *Ann Appl Biol* **136**: 167-172.

Schindelin J, Arganda-Carreras I, Fris E, Kaynig V, Longair M, Pietzsch T, Preibisch S, Rueden C, Saalfeld S, Schmid B, Tinevez JY, White DJ, Hartenstein V, Eliceiri K, Tomancak P, Cardona A. (2012). Fiji: an open-source platform for biological-image analysis. *Nat Methods* **9**: 676-82.

Toki S, Hara N, Ono K, Onodera H, Tagiri A, Oka S. Tanaka H. (2006). Early infection of scutellum tissue with Agrobacterium allows high-speed transformation of rice. *Plant J* **47:** 969-976.

VSN International (2019). Genstat for Windows 20th Edition. VSN International, Hemel Hempstead, UK. Web page: Genstat.co.uk

Zhong S, Lin Z, Fray RG, Grierson, D. (2008). Improved plant transformation vectors for fluorescent protein tagging. *Transgenic Res* **17**: 985-989.

Zhou H-Y, Songbiao Ch, Xugang L, Guifang X, Xiaoli W, Zhen Z. (2003). Generating marker free transgenic tobacco plant by Agrobacterium mediated transformation with double T-DNA Binary vector. *Acta Botanica Sinica* **45**: 1103-1108.
